# Digital Multidomain Lifestyle Intervention for Community-Dwelling Older Adults: A Mixed Methods Evaluation

**DOI:** 10.3389/ijph.2025.1608014

**Published:** 2025-03-14

**Authors:** Renato Mattli, Manuel Weber, Anja Maria Raab, Karin Haas, Albrecht Vorster, Kai-Uwe Schmitt

**Affiliations:** ^1^ Academic-Practice-Partnership between School of Health Professions at Bern University of Applied Sciences and University Hospital of Bern, Bern University of Applied Sciences, Bern, Switzerland; ^2^ Epidemiology, Biostatistics and Prevention Institute, University of Zurich, Zurich, Switzerland; ^3^ Swiss School of Public Health (SSPH+), Zurich, Switzerland; ^4^ Institute on Ageing, School of Health Professions, Bern University of Applied Sciences, Bern, Switzerland; ^5^ Swiss Sleep House Bern, Department of Neurology, University Hospital of Bern, University of Bern, Bern, Switzerland; ^6^ Interdisciplinary Sleep-Wake-Epilepsy-Center, University Hospital of Bern, University of Bern, Bern, Switzerland

**Keywords:** physical activity, nutrition, sleep, mindfulness, relaxation, lifestyle medicine, health-related quality of life, mHealth

## Abstract

**Objectives:**

As life expectancy rises at a faster rate than healthy life expectancy, there is a global need for scalable and cost-effective interventions that enhance the health-related quality of life of older adults. This study aimed to examine the user experience and usability of a 12-week digital multidomain lifestyle intervention in community-dwelling older adults aged 65 years and above.

**Methods:**

The intervention was developed involving older adults and delivered through a mobile application (app) focusing on physical activity, nutrition, sleep and mindfulness/relaxation. We used a mixed methods sequential explanatory approach to evaluate the user experience and usability of the intervention. We delivered online questionnaires before and after the intervention, collected app usage data and conducted semi-structured interviews.

**Results:**

One hundred eight older adults participated in the study. Fifty-six percent of participants completed the 12-week intervention. Users who completed the intervention experienced it as highly satisfactory and rated the usability as high. User engagement was particularly high for the physical activity content.

**Conclusion:**

Although participant retention can be a challenge, a digital multidomain lifestyle intervention developed involving community-dwelling older adults can lead to positive user experience and high usability.

## Introduction

Lifestyle behaviour contributes to healthy aging with lifestyle influencing health-related quality of life (HRQoL) and longevity [1]. Although life expectancy and healthy life expectancy are growing globally, healthy life expectancy is increasing at a lower rate than life expectancy [2]. A healthy lifestyle may extend healthy life expectancy, as a healthy lifestyle appears to be associated with an increase in the number of years lived without major chronic disease [3]. Furthermore, a healthy lifestyle, even in late life, can greatly mitigate the genetic risk of a shorter lifespan [4, 5]. Consequently, older adults should be enabled to adopt a healthy lifestyle.

Lifestyle medicine is an evidence-based discipline that follows a biopsychosocial approach, focusing on six key domains: sleep, nutrition, physical activity, stress, abuse of risky substances, and social relationships [6]. Lifestyle interventions that are based on the concept of lifestyle medicine place the individual at the centre and take a holistic view of their daily lives [7]. Multidomain lifestyle interventions (MLIs) address several domains as part of one intervention. In older adults, MLIs were shown to improve important dimensions of HRQoL [8], maintain daily functioning [9], and reduce cognitive decline [10–12], sarcopenia [13], inflammation levels [13] and the risk of developing new chronic diseases [14]. Self-efficacy for healthy lifestyle behaviour was also positively influenced by a lifestyle intervention [15]. Furthermore, a MLI was shown to be cost-effective in preventing dementia [16].

Most MLIs for older adults focused on two lifestyle domains, physical activity and nutrition [10, 17]. However, another lifestyle domain with an important contribution to healthy aging is sleep [18]. This domain has been less frequently included in MLIs [19]. Diverse sleep problems, including sleep apnea, insomnia, restless legs and excessive daytime sleepiness, show a high prevalence in older adults [20] and are associated with reduced health and quality of life [21, 22]. Furthermore, several lifestyle factors such as physical activity [23], nutrition [24], social relationships [25] and mindfulness [26, 27] seem to influence sleep quality in older adults. Mindfulness-based interventions also have the potential to positively influence quality of life and cognition in older adults [28–30]. Additionally, a recent qualitative review highlighted the development of new perspectives based on mindfulness-based interventions in older adults, including enhanced coping with negative situations, greater acceptance and an increased ability to focus on the present moment [31].

The two main modes of delivery for MLIs are face-to-face and digital [32]. Digital MLIs offer various advantages, including the flexibility to be used anytime and anywhere, personalization and low costs [19, 33]. These advantages address some important barriers and facilitators to implementing MLIs among community-dwelling older adults [34]. Digital MLIs are available in different formats, such as websites, mobile applications or a combination of both [19]. Most digital MLIs focus on the impact of measures including weight, body mass index (BMI), minutes of physical activity, daily step count or clinical parameters (e.g., blood pressure or cholesterol levels) but much less on HRQoL or mental wellbeing [33, 35]. However, HRQoL is an important patient reported outcome measure that is related to physical, mental and social aspects [36]. Mental wellbeing is another concept that is associated with HRQoL [37]. A recent study showed increases in domains of HRQoL and mental wellbeing with a 10-week digital MLI in a general adult population [38]. Furthermore, the effectiveness of digital MLIs on physical activity, nutrition, sleep and brain health outcomes in various populations has been shown in recent meta-analyses [19, 33]. In addition, there is evidence that older adults engage in digital mental wellbeing interventions [39]. However, there is a lack of digital MLIs targeting HRQoL and mental wellbeing that have been developed and evaluated involving older adults and address the sleep and stress domain [19, 33].

Therefore, this study aimed to examine the user experience and usability of a 12-week digital MLI that has been developed involving community-dwelling older adults aged 65 years and older, and incorporated four lifestyle domains (physical activity, nutrition, sleep and mindfulness/relaxation) to improve HRQoL and mental wellbeing.

## Methods

We conducted a mixed methods study using a sequential explanatory approach to further investigate and interpret quantitative results through qualitative data [40].

### Setting, Participants and Recruitment

Community-dwelling older adults aged 65 years and older who understand German were included. Furthermore, access to a smartphone or tablet (Android-Version 10 or Apple iOS-Version 13 or later) was an inclusion criterion. Existing disabilities or diseases were not used as exclusion criteria. The intervention was home-based, with no online or in-person meetings. In case of any questions, participants could contact the research staff via the contact form in the app or email.

We used chain referral sampling to recruit diverse older adults in the German-speaking region of Switzerland. The study invitation and the eligibility criteria were announced via print, email, social media, or on the corresponding website of the research project, Senior Citizens’ Universities, senior platforms and websites, senior associations, and service providers for older people in Switzerland. A link to a website with further information was embedded in this invitation. On this website, interested individuals could leave their contact details. An email with all the details of the study, including the instructions for installing the app, was sent to all interested individuals. Participation in the study was voluntary and participants could withdraw at any time without giving reasons. Nonparticipation did not entail any disadvantages. The respondents were assured pseudonymized data handling. Participants were recruited from August to November 2023.

### Digital Multidomain Lifestyle Intervention

**FIGURE 1:**
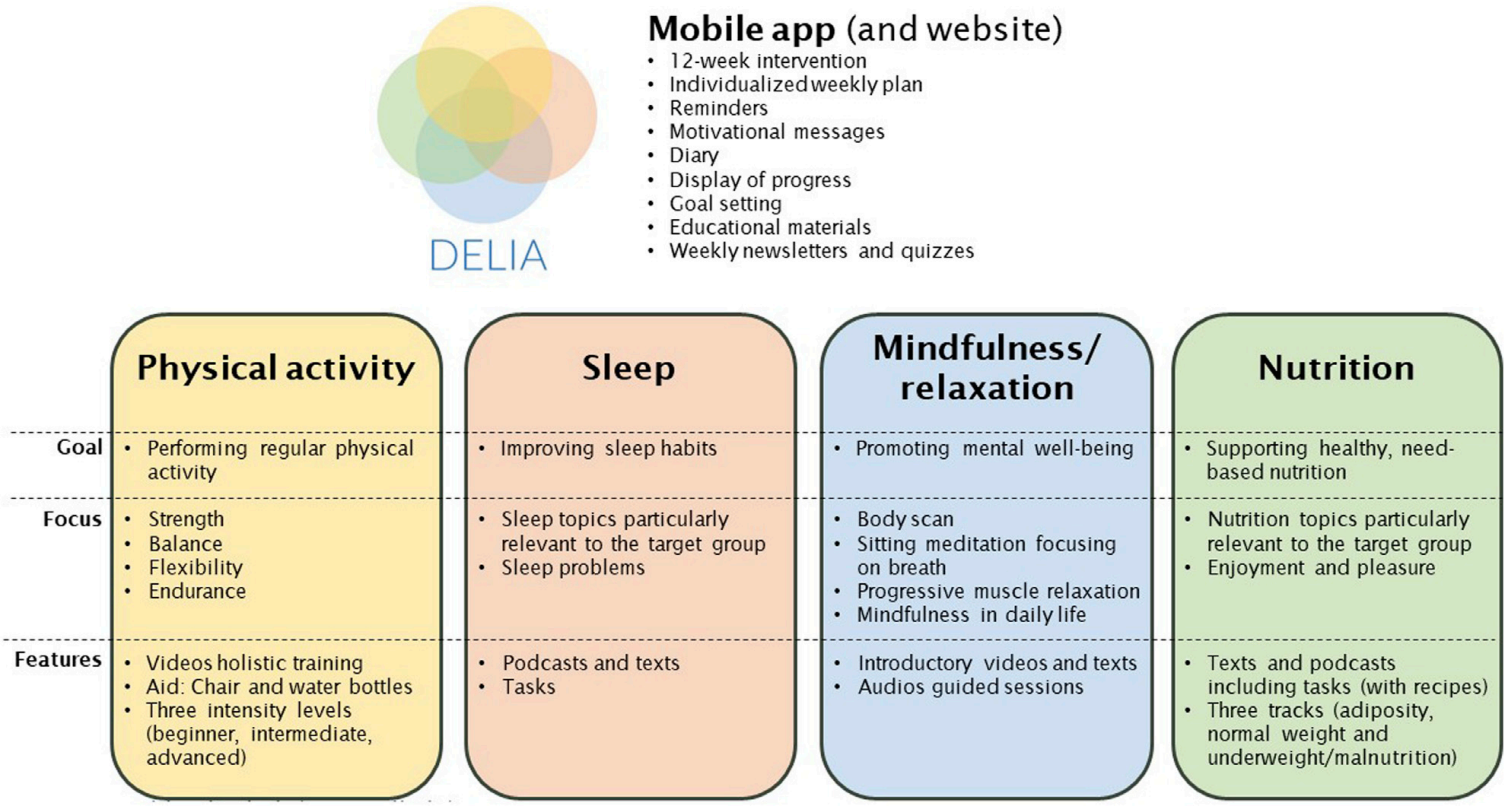
Overview of the app content (Switzerland, 2024).

In this project, we examined a 12-week digital MLI with four core domains: physical activity, nutrition, sleep and mindfulness/relaxation. The intervention was delivered through a mobile application. In parallel, a website containing all the intervention content was developed to allow viewing on larger screens and to enable offline access through downloads. Figure 1 gives an overview of the main content; details can be found in Supplementary Appendix Section S1.

The overall purpose of the intervention was to increase HRQoL and mental wellbeing by enabling and empowering older adults to cultivate a deliberate and healthy lifestyle. Physical activity and nutrition content was provided over 12 weeks, whereas the content for sleep and mindfulness/relaxation lasted 6 weeks. Although it has been recommended to extend the number of lifestyle domains included in digital MLIs [19], user engagement of such interventions depends on the relationship between perceived benefits and costs [41]. The more lifestyle domains a MLI covers, the higher the costs in terms of time required to spend on the MLI. Time constraints have been reported as a common reason for dropping out of digital MLIs [19], therefore, we wanted to keep the time required to spend on our digital MLI on a reasonable level and decided to provide the content for sleep and mindfulness/relaxation sequentially, both lasting 6 weeks, rather than in parallel. To investigate the user experience related to these two less common lifestyle domains, we used a cross-over interventional design in which group A received access to modules related to physical activity, nutrition, and mindfulness/relaxation during the first six weeks, whereas the module mindfulness/relaxation was replaced by sleep the following 6 weeks and *vice versa* in group B.

Each user had an individually tailored structured weekly schedule of activities based on the information entered at the beginning. In addition, both the content of the physical activity and nutrition domains were personalized. The physical activity domain consisted of a multicomponent exercise training twice a week and recommendations for endurance training twice a week. The exercise training comprised videos and focused on strength in the upper and lower limbs and core as well as balance and flexibility. One session lasted between 20 and 40 min. The endurance training started with a duration of 15 min and gradually increased to 45 min. Several sample aerobic activities were proposed and the recommended intensity followed aerobic training zones including rating of perceived exertion (5–6) (modified Borg CR10 Scale [42]). The nutrition domain offered information, advice and tips on nutrition in older age twice a week, each session lasting 5–25 min. The sleep domain provided knowledge, advice and guidance for improving sleep habits. Two sessions per week of 5–20 min each were scheduled. In addition, participants were asked to complete a sleep protocol for 2 weeks. The mindfulness/relaxation domain introduced participants to evidence-based stress management techniques that have been shown to enhance mental wellbeing [43, 44]. We included three techniques: body scan, sitting meditation focusing on breath and progressive muscle relaxation. Each technique was practiced for 2 weeks. Two sessions per week 20 min each were scheduled, but participants were informed that these techniques could be practiced more regularly. If participants completed a session, they were asked to mark it as complete in the app. In addition, the intervention included weekly newsletters and quizzes. The time required for the intervention varied from person to person, but it was approximately three to four hours per week. Participants were onboarded to the intervention via email. This email included detailed participant information, instructions on how to install the mobile application and a short overview of the main functions of the mobile application. In case of questions or issues, they could contact the study team via email or the app (details Supplementary Appendix Section S1).

Sustainable implementation has been identified as a key challenge for digital health interventions [19, 45] that may be successfully addressed by thoroughly involving end-users during the development and evaluation of such interventions [45, 46]. Therefore, we used a user-centred design approach to develop the intervention, meaning that an iterative design process involving end-users (i.e., older adults) was applied in the design of the digital MLI [47]. This can also be considered as a participatory co-creation approach [48] (details Supplementary Appendix Section S2).

Furthermore, we used a multidisciplinary approach [45] and involved experienced health professionals and researchers from various disciplines including sleep, nutrition and dietetics, physiotherapy, exercise and sports science, mindfulness, psychology, gerontology, and software development. In addition, our intervention was mainly based on the following behaviour change technique clusters [49]: goals and planning, feedback and monitoring, shaping knowledge, repetition and substitution, comparison of behaviour and natural consequences. The development of the interventional content was based on available literature (including previous research from the study team [50]) and current recommendations of national and international institutions (e.g., the Swiss Society for Nutrition and the World Health Organization).

### Mixed Methods

#### Quantitative Part

##### Assessments

All participants completed a self-administered online questionnaire before the start of the intervention. During the intervention, daily app usage data and participant feedback submitted via the contact form in the app or email were collected. After the intervention, participants were again asked to complete an online questionnaire. Furthermore, all participants who stopped using the app before the end of the intervention were contacted and asked to provide reasons for stopping in a short online questionnaire. All online questionnaires were created using LimeSurvey (LimeSurvey GmbH, Hamburg, Germany, Version 2.56.1).

##### Measures

Participant characteristics: We collected data regarding age, gender, height, weight, general health status using SF-36 [51], current satisfaction with each lifestyle domain (self-developed; 7-point Likert scale, 1 [very dissatisfied], 7 [very satisfied]) and readiness to change (based on [52]; 11-point Likert scale, 0 [not at all ready to change], 10 [very ready to change]).

User Experience: User experience was defined according to Wesselman et al. [19]. Daily app usage data was automatically tracked during the intervention. This means the app documented the date and time an app domain (i.e., weekly plan, diary, newsletter and quiz, progress, help and safety and settings) was visited, a participant marked a session as complete and a diary entry was made. Overall satisfaction with the app was assessed with the following two statements from the mHealth App Usability Questionnaire (see below): “Overall, I am satisfied with this app.” and “I would use this app again.” using a 7-point Likert scale ranging from 1 (fully disagree) to 7 (fully agree). In addition, participants who finished the intervention were asked if the app helped to move regularly/to eat healthy and according to their needs/to improve their sleep habits/to improve their mental wellbeing on a 7-point Likert scale ranging from 1 (fully disagree) to 7 (fully agree). These questions were self-developed and we used the Likert scale similar to the mHealth App Usability Questionnaire (see below). In addition, weekly overall self-reported health was assessed within the app using the EuroQol Visual Analogue Scale (EQ VAS) [53].

Usability: The app usability was assessed with the mHealth App Usability Questionnaire (MAUQ) for standalone apps after the intervention was completed [54]. This questionnaire has 18 statements, and each has to be rated on a 7-point Likert scale ranging from 1 (fully disagree) to 7 (fully agree). The MAUQ has three subscales: ease of use, interface satisfaction and usefulness. The German version of the MAUQ showed an internal consistency of Cronbach’s α = 0.93 and demonstrated high reliability [55].

##### Analysis

Questionnaire and usage data were summarized using numbers and percentages for qualitative variables, mean and standard deviation for quantitative variables with normal distribution and median as well as 25th/75th percentiles for quantitative variables with non-normal distribution.

#### Qualitative Part

##### Assessments

After the 12-week intervention, in-depth semi-structured interviews (one-on-one by phone) with randomly invited participants from the subgroup who completed the intervention were conducted in German until saturation was reached. Interviews were recorded in Microsoft Teams (Microsoft, Redmond, United States, Version 2023.38.01.50).

##### Measures

We developed an interview guide exploring user experience, usability, and ideas for improvement (details Supplementary Appendix Section S3.).

##### Analysis

The interviews were transcribed verbatim and a thematic analysis was performed [56]. Each interview transcript was first read in its entirety. They were then re-read and initially coded using comparative methods also considering quantitative results. In the next step, specific research team meetings were scheduled to review the initial coding. During these meetings, we focused on frequent initial codes or codes with a high significance for the research topic, searched for relationships between the initial codes, connected them and created categories.

#### Data Integration of Quantitative and Qualitative Parts

The quantitative and qualitative parts were connected at the intermediate stage, where the results of the data analysis of the quantitative part informed and guided the data collection of the qualitative part [57]. At the interpretation and reporting level, data integration and presentation followed the four-stage technique of the Pillar Integration Process to get to a joint display of quantitative and qualitative findings [58].

### Pre-Post Comparison of Effectiveness Measures

To investigate the potential effectiveness of the intervention on important dimensions of HRQoL and mental wellbeing, we used the mental health and vitality subscales from the SF-36 [51] and the flourishing scale [59]. The SF-36 has been recommended particularly in community-dwelling older adults with limited morbidity to assess detailed aspects of HRQoL [60]. SF-36 subscales range from 0 to 100, where 100 represents maximum mental health and vitality, respectively. The flourishing scale has been used in a recent study investigating the effectiveness of a digital MLI for adults [61] and the scale ranges from 8 (minimum) to 56 (maximum flourishing). Changes from pre-to post-intervention were analysed using paired t-tests (adjusted for multiple comparisons) in the subsample of participants who used the app until the end (12 weeks) and completed the post-intervention questionnaire (n = 57). P values <0.05 were considered statistically significant. Analyses were conducted using R software (version 4.3.3 for Windows).

### Sample Size

Based on the sample size of quantitative studies investigating similar interventions [62–64], our experience from recruiting for a needs assessment for digital lifestyle interventions in Swiss community-dwelling older adults [50] and the planned duration of the recruitment phase (4 months) the target total sample size was 100 participants with 50 in each group.

## Results

### Participants

**FIGURE 2:**
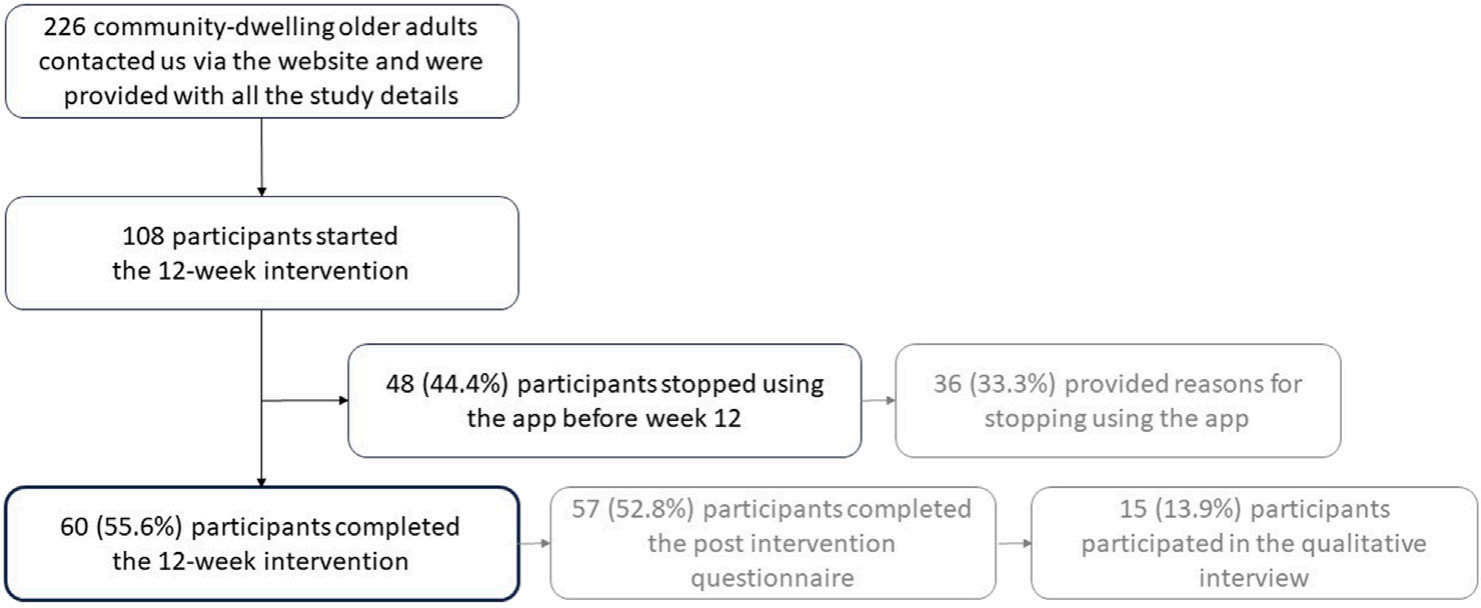
Participant flow-chart (Switzerland, 2024).

One hundred eight community-dwelling older adults participated in the study, 52 in group A and 56 in group B (Figure 2). Interviews were conducted with 15 participants and the interviews lasted between 20.4 and 42.0 min (mean 32.2 min, SD 6.8 min). Key baseline characteristics of the participants are shown in Table 1; details can be found in Supplementary Appendix Section S4.1. The most common reasons for study participation were an interest in lifestyle and wellbeing (seven interviewees), curiosity about the app (four interviewees) and an interest in technical aspects (two interviewees).

**TABLE 1:** Baseline characteristics (n = 108) (Switzerland, 2024).

Item	Mean (standard deviation); n (% of total population)
Sociodemographic	
Age (years)	71.1 (5.7); range: 64–98
Gender	
Female	67 (62.0%)
Male	41 (38.0%)
General health	
SF-36 general health status	
Excellent	3 (2.8%)
Very good	54 (50%)
Good	49 (45.4%)
Less favourable	2 (1.8%)
Poor	0 (0%)
Body mass index	24.6 (4.9); range: 17.2–51.4
Domains of health-related quality of life and mental wellbeing	
SF-36 mental health (0–100)	78.4 (12.4); range: 36–100
SF-36 vitality (0–100)	67.6 (14.7); range: 15–100
Flourishing scale (8–56)	46.8 (4.6); range: 32–56
Lifestyle	
Current satisfaction (1 [very dissatisfied] – 7 [very satisfied])	
Physical activity	5.4 (1.3); range: 2–7
Nutrition	5.5 (1.3); range: 1–7
Sleep	5.2 (1.5); range: 1–7
Mindfulness/relaxation	5.1 (1.4); range: 1–7
Readiness to change (0 [not at all ready to change] – 10 [very ready to change])	
Physical activity	8.2 (1.9); range: 0–10
Nutrition	7.8 (2.0); range: 0–10
Sleep	7.5 (2.6); range: 0–10
Mindfulness/relaxation	8.0 (2.2); range: 0–10

### User Experience

Detailed results from both the quantitative and qualitative research, data integration and pillar building themes are presented in Table 2.

**TABLE 2:** Data integration (Switzerland, 2024).

Quantitative data (n = 57; post-intervention questionnaire and app usage data)	Quantitative category	Pillar building themes	Qualitative category	Qualitative codes (n = 15, interviews)
**Overall satisfaction**				
“Overall, I am satisfied with this app.” (MAUQ) mean 6.0 (SD 1.0) “I would use this app again.” (MAUQ) mean 5.7 (SD 1.5)	High overall satisfaction and participants would use the app again	High overall satisfaction with high likelihood of using the app again, recommending the app to others and continue using the app	Would recommend the app to others (14/15) Would like to continue using the app (11/15)	“I would definitely recommend it [the app]. Absolutely.” (#08, 00:20:58) I hope it [the app] will still be available to me later.” (#03, 00:06:53)
General aspects liked the most: Variety of topics (11/57) Holistic approach to lifestyle (6/57) Simple handling (5/57) Inspiration (5/57) Structure of the app (4/57) App structured the days and weeks (3/57) Flexibility/no pressure (3/57) App was motivating (3/57)	General aspects that were liked most include the variety of topics, holistic approach to lifestyle, simple handling, structure of the app, app structured the days and weeks, inspiration and flexibility/no pressure	General aspects that were liked most include the variety of topics, holistic approach to lifestyle, simple handling, structure of the app and app structured the days and weeks	General aspects liked the most: Variety of topics (4/15) Holistic approach to lifestyle (3/15) Simple handling (3/15) Structure of the app (3/15) App structured the days and weeks (2/15)	“I actually liked the diversity, that is, many topics that concern us in old age, such as mindfulness, sleep and nutrition.” (#04, 00:00:48) “[…] especially how it all comes together nicely. It stands on its own, but there really is a bigger picture then.” (#09, 00:19:29) “What I liked best was actually the simplicity of operation, quite clear about what one could enter, what one could operate, what one could record.” (#12, 00:01:18) “For me, a structured program has been good. […] I had an overview of the week, knowing what I really needed to do, in the sense that I was guided a bit.” (#10, 00:02:52)
**Physical activity domain**				
Sessions completed (app usage data): - Holistic training: mean 88% (SD 55%) - Aerobic training: mean 104% (SD 54%)	Physical activity sessions highest completion rate (some participants made more than intended by the program; aerobic training higher than holistic training; high variability between participants)	Physical activity sessions with very high completion rate in a population with already high activity level and although some instructions were considered as not being helpful	Aerobic training: - Instructions and tasks most helpful (2/15) - Instructions and tasks NOT helpful (4/15) - Recommendation regarding intensity not fully clear (2/15) Already very active person (3/15)	“That [instructions for aerobic training] did not help me very much. There were practically no instructions.” (#11, 00:10:53) “What does intensive mean? […] Heart rate? […] I had for example, no clue there. I was simply told to go for a walk, it should be intensive, about a five [Borg Scale].” (#01, 00:32:33) “[…] regarding the physical aspect, it was not challenging enough for me because I do something every day, at least every other day.” (#15, 00:02:18)
Most helpful content: - Videos holistic training (7/57) Continue to continue use: - Videos holistic training (17/57)	Holistic training videos most helpful content in physical activity domain and content participants would like to continue to use	Holistic training videos most helpful content in physical activity domain and content participants would like to continue to use	Most helpful content: - Videos holistic training (10/15) Physical activity content motivated participants to try out new things (2/15) Continue to use: - Videos holistic training (2/15)	“Yes, these video sequences were of course very helpful. You could see it right away.” (#11, 00:09:40) “I also liked to participate [in the physical activity domain] and I also got new impulses from it.” (#08, 00:03:47)
“The holistic training was not challenging enough.” (self-developed; 7-point Likert scale) mean 4.8 (SD 1.7) “The increase in intensity of the holistic training was too low over the 12 weeks.” (self-developed; 7-point Likert scale) mean 4.8 (SD 1.6)	Intensity of holistic training appropriate or slightly too low Increase of intensity of holistic training appropriate or slightly too low	The intensity of the holistic training needs to match the individual level and increase over the time course of the program	Higher intensity preferred (2/15)	“So, a bit more rigorous from the beginning, right? Because you want to be somewhat challenged.” (#09, 00:25:19)
“The app helps me to move regularly.” (self-developed; 7-point Likert scale) mean 5.3 (SD 1.5)	Content of physical activity domain helped participants to move regularly	Physical activity content helped to move regularly (self-reported: more regularly and more mindful)	Physical activity content influenced participants at least partially (9/15): - More mindful physical activity (3/15) - Being more regularly active (2/15)	“[…], I have been aware that I am more mindful during movements, […].” (#14, 00:07:16) “[…], I have been on it [physical activity] much more regularly than I usually am, […].” (#13, 00:10:35)
**Nutrition domain**				
Sessions completed (app usage data): mean 65% (SD 25%)	On average 65% of nutrition sessions completed (high variability between participants)	Nutrition sessions with satisfactory completion rate and high variability between participants Nutrition content perceived as helpful although it did not provide new information to everyone Confirming what is already known about diet can act as a reminder Texts and podcasts are helpful modes of delivery	Most helpful content: - Content educational although a lot of information was not totally new (5/15) - Content confirmed what is already known and acts as reminder (5/15) - Content regarding a varied and balanced diet (2/15) - Content regarding proteins (2/15) There was no content related to nutrition that was reported as not being helpful during the interviews Nutrition content motivated participants to try out new things (3/15) Mode of delivery: - Texts helpful (4/15) - Podcasts helpful (3/15)	“I have once again become aware of how diverse one should and can eat.” (#03, 00:07:45) “In today’s time, we hear a lot in this context. We talk a lot about health, healthy nutrition, exercise, etc. We know a lot, but nevertheless, we occasionally need to become aware of it again.” (#02, 00:16:58) “The information about proteins and vitamin D was helpful, […].” (#14, 00:09:11) “I always eat the same thing for breakfast and the same thing for dinner. Every day. And of course, the program has already given me ideas about what else I could eat.” (#11, 00:15:10)
“The app helps me to eat healthy and according to my needs.” (self-developed; 7-point Likert scale) mean 5.1 (SD 1.3)	Content of nutrition domain helped participants to eat healthy and according to their needs	Content of nutrition domain helped participants to eat healthy and according to their needs (self-reported: more mindful, eat slower and drink more)	Nutrition content influenced participants (8/15): - More mindful eating (3/15) - Eating slower (2/15) - Drinking more (2/15)	“I want to feel good, and that is an important point, to consider nutrition.” (#08, 00:08:54) “[…] in the end, I prepared a plate for myself. We usually eat well, but I always thought, do I have the three components on it? The information was so persistent that it stuck with me.” (#13, 00:01:46) “When we were together, we have paid attention to eating slowly, enjoying, and doing the whole thing more consciously.” (#02, 00:19:50) “Eating more fruits. And drinking more.” (#11, 00:15:26)
**Sleep domain**				
Sessions completed (app usage data): mean 56% (SD 32%); group A: 49%; group B: 64%	On average 56% of sleep sessions completed (high variability between participants) Completion rate higher in the group that started with the sleep domain compared to the group that started with the mindfulness/relaxation domain	Sleep sessions with satisfactory completion rate and high variability between participants Completion rate higher if content at the beginning of the intervention Sleep content in general perceived as important and useful	Most helpful content: generally perceived as important and useful because: - New or insightful information (5/15) - Information that confirmed what they already knew (4/15) - Podcasts (4/15) Sleep content motivated participants to try out new things (5/15)	“It was helpful to see how much I actually sleep on average. I thought it was less, so I was positively surprised that I sleep for so long.” (#14, 00:18:13) “Yes, there [sleep domain] I have the feeling that I have learned the most.” (#05, 00:18:18) “Yes, the thing with sleep, the thing with the sleep log, that was something new for me and I tried it and found it very interesting.” (#07, 00:06:58)
Continue to use sleep content (10/57)	Sleep domain provided content that participants would like to continue to use	Sleep domain provided content that participants would like to continue to use	Continue to use (2/15)	“I will definitely do the sleep log at some point. I have quite a bit of potential for improvement there […].” (#05, 00:08:04)
“The app helps me to improve my sleep habits.” (self-developed; 7-point Likert scale) mean 4.9 (SD 1.5)	Content of sleep domain helped participants to improve sleep habits	Sleep content helped to improve sleep habits (self-reported: be more conscious about sleeping behavior, drink no coffee in the evening and be more relaxed when waking up during the night)	Sleep content influenced participants (8/15): - More conscious about the sleeping behavior (2/15) - No more coffee in the evening (2/15) - More relaxed when waking up during the night (2/15)	“It made me more relaxed. Before, it sometimes stressed me out when I could not sleep.” (#03, 00:10:50) “When I wake up very early in the morning and cannot fall back asleep, I do not think ‘Oh no, why now.’ Instead, I try to accept it. That has already changed.” (#07, 00:16:06) “Omitting coffee has helped, I stayed awake less often or had the feeling that I was awake. I could fall asleep faster.” (#14, 00:19:15)
**Mindfulness/relaxation domain**				
Sessions completed (app usage data): mean 52% (SD 32%); group A: 55%; group B: 49%	On average 52% of mindfulness/relaxation sessions completed (high variability between participants) Completion rate higher in the group that started with the mindfulness/relaxation domain compared to the group that started with the sleep domain	Mindfulness/relaxation sessions with satisfactory completion rate and high variability between participants Completion rate higher if content at the beginning of the intervention Audio files that guided through the different mindfulness/relaxation techniques most helpful Choice of different voices for audio files preferred	Most helpful content: - Audio files that guided through the different mindfulness/relaxation techniques (7/15) Mindfulness/relaxation content motivated participants to try out new things (4/15) Disliked voice in audio files (2/15)	“They [audio files] were good. […] I just thought to myself, it’s actually quite nice to be verbally guided.” (#06, 00:16:37) “So, in some things, that was very motivating. For example, with these exercises, like the body scan and these meditation exercises. I really liked them.” (#11, 00:06:22) “What I found difficult was the topic of mindfulness and relaxation. Just this kind of person who conducted the first sequence, I did not find their voice pleasant.” (#03, 00:02:36)
Continue to use mindfulness/relaxation content (15/57)	Mindfulness/relaxation domain provided content that participants would like to continue to use	Mindfulness/relaxation domain provided content that participants would like to continue to use	Continue to use mindfulness/relaxation content (2/15)	“In that sense, I do have the feeling to regularly incorporate the topic of mindfulness again. (#03, 00:06:27)
“The app helps me to improve my mental wellbeing.” (self-developed; 7-point Likert scale) mean 5.1 (SD 1.3)	Content of mindfulness/relaxation domain helped participants to improve mental wellbeing	Content of mindfulness/relaxation domain helped participants to improve mental wellbeing (self-reported: be more mindful and more relaxed)	Mindfulness/relaxation content influenced participants (9/15): - Being more mindful (3/15) - Being more relaxed (2/15)	“With more mindfulness, contentment, that helped me a lot, I changed the most there. And then also in connection with breathing, with breathing exercises, I notice that it is very good for me.” (#02, 00:15:37) “I think I received a lot of inputs. ‘Oh, I could think about that again’ or awareness, that is really important or something like that.” (#04, 00:16:41)
**Newsletter**				
Out of twelve newsletters, participants read on average 10.9 (SD 2.3); self-reported in the post-intervention questionnaire	On average eleven out of twelve newsletters read	Very high number of newsletters read (self-reported) Newsletter helpful because repetition of important aspects in a compact and informative way, confirmation what is already known and possibility to print out	Most helpful aspects: - Confirmation of what is already known (4/15) - Repetition of important aspects in a compact and informative way (3/15) - Two participants printed the newsletters	“Yes, I actually found them [newsletters and quizzes] very good. […] many times, when I was in the car with my husband, I read something from it to him again. I actually found it very informative. And, yes, when you are of a certain age, you already know so much and yet it feels good to become aware of it again, […].” (#04, 00:06:07) “There was nothing really new in any domain, but it is a confirmation of the importance and I find that good and exactly because it comes so persistently again and again over 12 weeks, that is why it sticks with you.” (#13, 00:09:55)
**Quiz**				
Out of eleven quizzes, participants solved on average 9.4 (SD 3.1); self-reported in the post-intervention questionnaire	On average nine out of eleven quizzes solved	High number of quizzes solved (self-reported) Ambivalent perception of quizzes. On one hand, they were perceived as informative and good as well as a confirmation of what they had read. On the other hand, they were perceived as too simple and not motivational	Perceived as informative and good as well as a confirmation of what they have read (4/15) Perceived as too simple and not motivational (3/15)	“Yes, very simple, I knew all of that, if you read the newsletter, it is like a confirmation that you understood it.” (#14, 00:06:30) “I have read it [quiz] through. But I did not have much motivation for it.” (#08, 00:03:22)

#### Participant Retention

**FIGURE 3:**
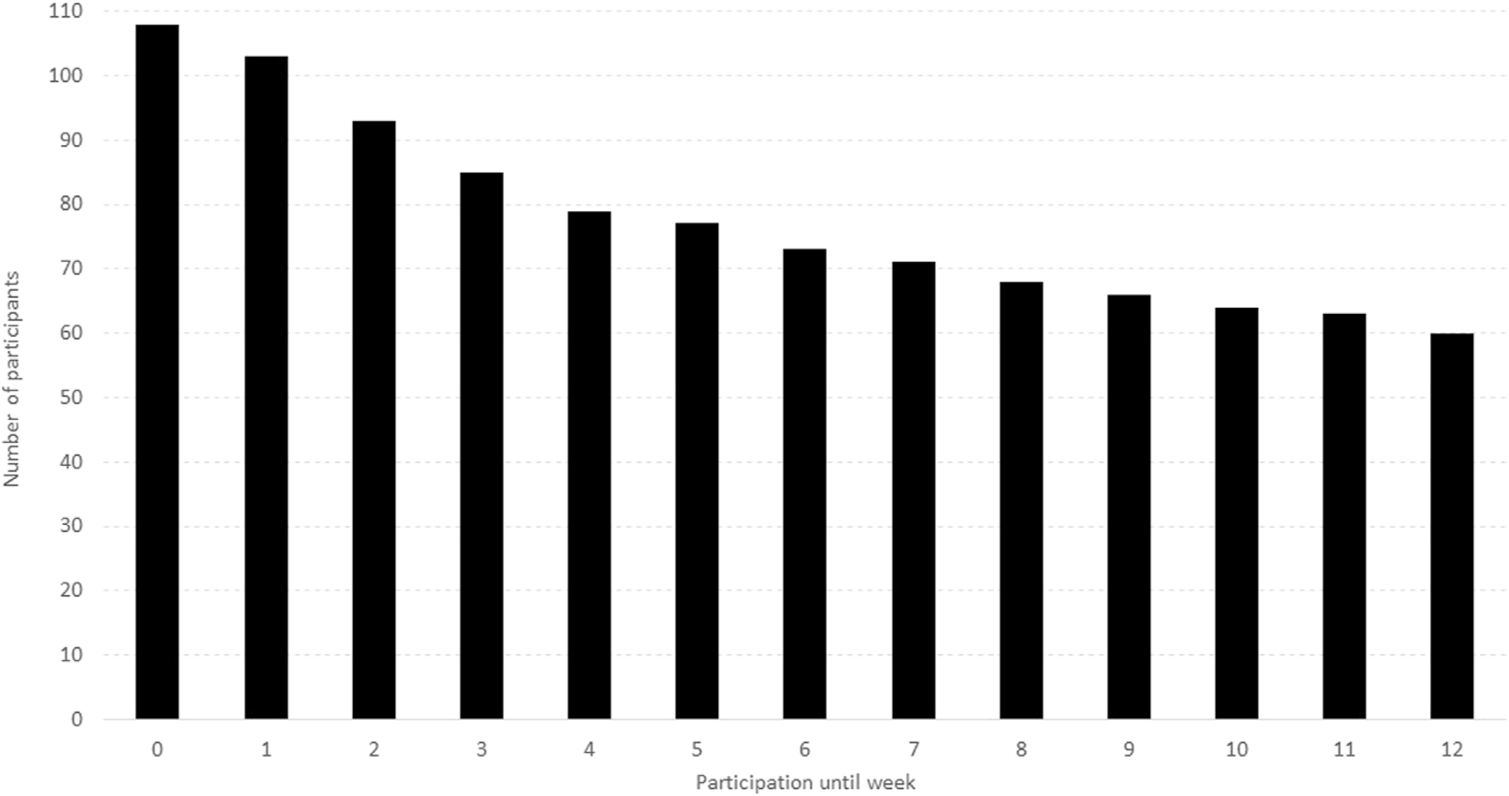
Duration of app use (Switzerland, 2024).

According to the app usage data, 60 participants (55.6%) used the app until week twelve (Figure 3). Of the ones who stopped earlier, 48% stopped using it within 2 weeks or even before the intervention started as five participants installed the app but did not start (“participation until week 0”).

From the 48 participants who did not complete the intervention, 36 participants (75.0%) provided some insights into why they stopped using the app. The reason most often mentioned was lack of time (9 participants) followed by too boring (5 participants), illness (4 participants) and injury, technical issues or holistic training exercises too easy (each mentioned by 3 participants).

The participants who did not complete the intervention tended to be older than the ones using the app until week twelve (71.8 years vs. 70.6 years) and more male than female participants stopped before week twelve (51.2% vs. 40.3%). Furthermore, these participants had on average a higher BMI (25.8 vs. 23.5) and the percentage of participants with a general health state of “excellent” or “very good” was lower (47.9% vs. 56.7%). Details can be found in Supplementary Appendix Section S4.3.

#### User Engagement

The app domain that was most often visited by the participants was weekly plan (50.7% of all visits) followed by diary (21.7%), newsletter and quiz (18.2%), progress (5.9%), help and safety (2.6%) and settings (0.9%). There was a total of 15,050 visits to the different domains of the app. Considering the duration participants used the app, this makes approximately 2.5 visits per participant per day.

On average, participants completed 104% (SD 54%) of the aerobic training sessions (it was possible to complete more sessions than those foreseen by the intervention), 88% (SD 55%) of the holistic exercise sessions, 65% (SD 25%) of the nutrition sessions, 56% (SD 32%) of the sleep sessions and 52% (SD 32%) of the mindfulness/relaxation sessions.

The sleep session completion rate was higher in group B that started with the sleep content in the first six weeks (64% vs. 49% in group A). Furthermore, group B rated the statement “I would have preferred to start with mindfulness/relaxation instead of sleep” with a mean of 3.2 (SD 1.3) on a 7-point Likert scale ranging from 1 (fully disagree) to 7 (fully agree). Similarly, the mindfulness/relaxation session completion rate was higher in group A that started with the mindfulness/relaxation content in the first six weeks (55% vs. 49% in group B) and group A rated the statement “I would have preferred to start with sleep instead of mindfulness/relaxation” with a mean of 2.9 (SD 1.5).

#### Overall Satisfaction

Respondents of the post-intervention questionnaire reported high overall satisfaction with the app (“Overall, I am satisfied with this app.” mean 6.0 (SD 1.0) on a 7-point Likert scale ranging from 1 (fully disagree) to 7 (fully agree)) and a high likelihood of using the app again (“I would use this app again.” mean 5.7 (SD 1.5)). Furthermore, 14 of 15 interviewees would recommend the app to others and eleven would continue using the app. General aspects that were liked the most included variety of topics addressed in the intervention (4/15 interviewees), holistic approach to lifestyle (three interviewees), simple handling of the app (three interviewees), structure of the app (three interviewees) and the fact that the app structured the days and weeks (two interviewees).

#### Most Helpful Content

According to the interviewees, the most helpful content in the physical activity domain was the exercise videos (10/15 interviewees) including the fact that no special aids or tools were required to perform the exercises (two interviewees). Five of 15 interviewees perceived the nutrition content as educational although a lot of information was not new to them. Furthermore, five interviewees liked that the content confirmed what they already knew and perceived it as a welcomed reminder. Text (four interviewees) and podcasts (three interviewees) were considered helpful modes of delivery. Furthermore, the sleep content was perceived as important and useful due to new or insightful information provided (5/15 interviewees), or confirming their previous knowledge (four interviewees). Seven of 15 interviewees perceived the audio files that guided through the different mindfulness/relaxation techniques as being the most helpful content.

#### Potential for Future Development

Five of 15 interviewees did not like the voice that guided the body scan and the sitting meditation. Four interviewees considered the aerobic training instructions as not helpful because they perceived them as not specific enough or not completely clear. Technical issues related to watching the exercise videos were mentioned by four interviewees; to some extent, however, the issues were due to poor internet connectivity of the users. A larger repertoire of exercises, along with the option to access content in offline mode, was desired by two interviewees. Two participants also suggested exercise videos with more pep and drive. Eleven interviewees said there was no content where they would have preferred to have a contact person for face-to-face interaction. The interviewees were also asked if they could think about using the app while being outdoors: six interviewees said yes, five no and four were uncertain. Furthermore, the following domains were reported to contribute to their mental wellbeing and could be considered as potential additions to the app by at least two participants of the post-intervention questionnaire: creativity (music, singing, painting), cognitive training, social relationships, faith/spirituality/religion, and nature.

#### Achieving Intended Goals

Participants rated the statement “the app helped me to move regularly” with a mean of 5.3 (SD 1.5; 7-point Likert scale). The statement “the app helped me to eat healthy and according to my needs” achieved an average rating of 5.1 (SD 1.3). The statement “the app helped me to improve my sleep habits” was rated with a mean of 4.9 (SD 1.5) and “the app helped me to improve my mental wellbeing” achieved an average rating of 5.1 (SD 1.3).

#### Influence on Behaviour

Nine interviewees reported that the intervention influenced their physical activity behaviour at least partially with more mindful physical activity being most often mentioned (three interviewees). In addition, eight interviewees reported that their behaviour related to eating was influenced with more mindful eating being most often mentioned (three interviewees). Eight interviewees reported the intervention impacted their sleep behaviour with more consciousness about sleeping behaviour, not drinking coffee in the evening and being more relaxed when awaking during the night each mentioned by two interviewees. In addition, nine interviewees reported that the mindfulness/relaxation content influenced their behaviour with increased mindfulness (three interviewees) and relaxation (two interviewees) being most frequently mentioned.

### App Usability

The overall app usability assessed with the MAUQ was on average rated at 5.6 (SD 0.7). From the three MAUQ subscales, ease of use was scored the highest (mean 6.0, SD 0.9), followed by interface satisfaction (mean 5.8, SD 0.7) and usefulness (mean 5.1, SD 0.9). Details can be found in Supplementary Appendix Section S4.5.

### Pre-Post Comparison of Effectiveness Measures

The SF-36 mental health subscale and flourishing scale showed a statistically significant change from pre-to post-intervention whereas the change for the SF-36 vitality subscale was not statistically significant. Details can be found in Supplementary Appendix Section S4.6.

## Discussion

We examined the user experience and usability of a 12-week digital MLI that has been developed involving community-dwelling older adults aged 65 years and older, and incorporated four lifestyle domains (physical activity, nutrition, sleep and mindfulness/relaxation) to improve HRQoL using a mixed methods approach. One hundred eight older adults participated in the study. Fifty-six percent of participants completed the 12-week intervention that was delivered through a mobile app. Users who completed the intervention experienced it as highly satisfactory and rated the usability as high. Furthermore, user engagement was particularly high for the physical activity content.

Participant retention has been described as a common challenge of digital health interventions [65]. For web-based interventions promoting health through behavioural change, approximately 50% of the users stopped before the end of the intervention [66]. Similarly, a recent scoping review found a median completion rate of remote digital health studies of 48% [67]. The dropout rate of 44.4% in our study is at the upper end of the range of 2%–52% reported in a recent meta-analysis of web-based MLIs for brain health in older adults [19]. We tried to reduce the complexity of tasks required from the participants and included regular reminders as nudges [67]. However, our intervention did not include personal contact for participants although this aspect may increase participant retention [67]. Especially, an in-person onboarding process may would have increased participant retention [67]. Although adherence to digital interventions may be increased with human support [68], the combination of digital and human support has its own challenges [41].

To enhance user experience of future interventions and identify potential barriers, we tried to gain insights into the reasons for not completing our intervention. By targeting a low burden for the respondents, we were able to receive feedback from 75% of the participants who did not complete the intervention. Reported reasons for dropping out were comparable to the results of a recent meta-analysis and included time constraints, physical illness, technical issues and dissatisfaction with the content [19]. The average participant who dropped out of our intervention tended to be older, had a higher BMI and reported a lower general health state than the ones who completed the intervention. Consequently, we may have not been able to satisfy the needs of some older and less healthy participants. As the health state is related to health literacy and digital health literacy [69, 70], this may also indicate that health literacy and digital health literacy was lower in the participants who did not complete the intervention. Therefore, there seems to be no one-size-fits-all solution for digital MLIs even if developed in a user-centred approach.

User engagement was especially high in the physical activity domain where participants on average completed more than the number of intended aerobic training sessions and 88% of the holistic exercise sessions. This corresponds to findings from a MLI with digital elements for improving brain health, where participants prioritized content topics according to the following order (from top to bottom priority): physical activity, cognitive training, nutrition, stress management, sleep, and social engagement [63]. Consequently, physical activity seems to be a core domain of MLI for older adults. In addition, the exercise videos were perceived as the most helpful content in the physical activity domain by ten out of 15 interviewees. This may be attributed to several intervention characteristics, such as its structured multicomponent design – incorporating strength, balance and flexibility training for the whole body (lower limbs, core and upper limbs) – and its three intensity levels which allowed for personalization. Additionally, the exercises were easy to perform at home or in other settings, requiring only a chair and additional weights (e.g., water bottles), and were demonstrated by peers.

In our MLI, we specifically added the two lifestyle domains sleep and mindfulness/relaxation to the more common domains of physical activity and nutrition. Further aspects such as social relationships, risky substance abuse and cognitive training, were also covered, but less extensively. Many interviewees particularly appreciated this holistic approach and the variety of topics covered. Furthermore, these lifestyle domains are interconnected. For example, our study showed the effect of the mindfulness/relaxation domain on the physical activity and nutrition domain with more mindful physical activity and more mindful eating being most often mentioned by the interviewees regarding how the intervention influenced their behaviour. This increased awareness of a lifestyle behaviour may change the type of motivation and lead to behaviour change [71, 72]. Consequently, the stress management lifestyle domain may act as a door opener for healthy behaviour in other lifestyle domains [73]. Similarly, the sleep domain is highly interconnected with the other five lifestyle domains and sleep influences goal-directed and stimulus-driven behaviour [74]. However, the benefit of a holistic approach and a variety of topics needs to be well balanced against the time required to spend on an MLI. Although we intended to gain insights into the impact of timing and order of the two additional domains sleep and mindfulness/relaxation using a cross-over design, the user engagement data did not reveal any substantial differences between the two groups. Therefore, giving choices to the users in regard to timing and order may be an option to further increase user engagement in future digital MLIs for older adults [41].

The usability observed in our study was slightly lower compared to an app specifically developed for patients with inflammatory arthritis [75] but higher than in a study investigating an mHealth app for patients with or at risk for cardiovascular disease [76]. Our app achieved the highest usability ratings for the two statements in the MAUQ “The app was easy to use.” and “It was easy for me to learn to use the app.” This might be largely due to our very simple and minimalistic app design using high contrast colours and allowing for large font sizes (which could be tailored).

A main strength of our study is the mixed methods approach, which enabled us to complement quantitative with qualitative data. This deepened our understanding of digital MLIs for community-dwelling older adults and provided guidance for future development and implementation [41].

As a limitation, our population was more vital than the average Swiss person in the same age group [77] and showed high physical activity levels at pre-intervention. Furthermore, we did not collect information about participants’ education, socioeconomic status or digital literacy. As a further limitation, abuse of risky substances was not addressed as a single domain but covered in the sleep domain (sleeping pills, alcohol, nicotine) and nutrition domain (alcohol). In addition, we did not include social relationships as an independent single intervention domain but rather addressed social aspects, for example, group physical activity, eating together or mindfulness communication, in the other domains to highlight the interconnection between the different lifestyle domains. However, participants mentioned that social relationships could be a more prominent topic in future MLIs. This also corresponds to previous findings showing that social aspects are a facilitator for older adults to participate in MLIs [34]. Therefore, future studies may investigate the user experience and usability of blended MLI approaches for community-dwelling older adults combining digital with face-to-face intervention activities specifically enabling social contact [66].

### Conclusion

This study aimed to examine the user experience and usability of a 12-week digital MLI to improve HRQoL in community-dwelling older adults aged 65 years and above using a mixed methods approach. The intervention was developed involving older adults and delivered through a mobile application focusing on physical activity, nutrition, sleep and mindfulness/relaxation. Although participant retention can be a challenge, our study shows that such a digital MLI can lead to positive user experience and high usability in community-dwelling older adults. These findings may inform the development and evaluation of future digital MLIs targeting HRQoL and mental wellbeing in older adults.
