# Supplementary material for: Digital Multidomain Lifestyle Intervention for Community-Dwelling Older Adults: A Mixed Methods Evaluation
Source: Int J Public Health. 2025 Mar 14;70:1608014. doi: 10.3389/ijph.2025.1608014 (PMC11949752; doi:10.3389/ijph.2025.1608014)
Supplement: Supplementary file 1 [file Presentation1.pdf]

APPENDIX to "Digital multidomain lifestyle intervention for community-dwelling older adults: A mixed methods evaluation "

Content

1. Detailed description of the digital multidomain lifestyle intervention 2
   1.1. Physical activity 3
   1.2. Nutrition 4
   1.3. Sleep 5
   1.4. Mindfulness/relaxation 6
   1.5. Newsletter 7
   1.6. Quiz 8
   1.7. Personalization 9
   1.8. Additional features 11
   1.9. Participant onboarding 12
2. App development 13
3. Interview guide 14
4. Additional results 22
   4.1. Baseline characteristics of the study population 22
   4.2. Weekly EQ VAS 23
   4.3. Non-completers versus completers – Additional aspects 24
   4.4. Newsletter and quiz 24
   4.5. App usability (MAUQ) – Detailed results 25
   4.6. Pre-post comparison of effectiveness measures – Detailed results 26
5. References 29

1. Detailed description of the digital multidomain lifestyle intervention

The name of the app was DELIA (Digital Lifestyle Intervention for Aged adults). The home screen and an example of the daily activities from the weekly plan are presented in Figure 1.

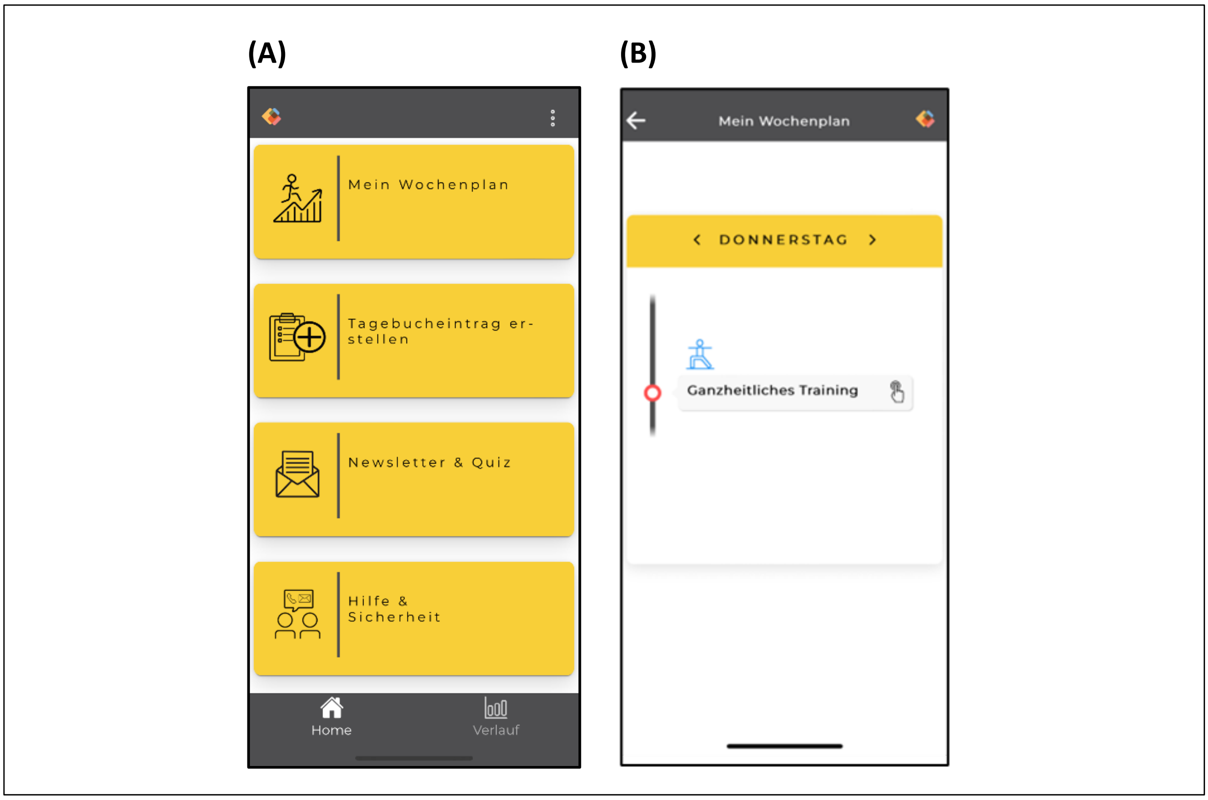

Figure 1: Screenshots of the application: (A) home screen, (B) overview of the daily activities as part of the weekly plan (Switzerland, 2024)

1.1. Physical activity

The physical activity domain consisted of instructional holistic exercise training videos focusing on full-body strength, balance, and flexibility (Figure 2). Exercises covered the upper limbs, lower limbs, and core. Each session included a full video for warm-up (5 min), followed by whole-body exercise training (15–45 min), and concluded with a cool-down involving three stretching exercises (5–10 min). Participants were able to easily perform the exercises at home or anywhere else, requiring only a chair and additional weights (e.g., water bottles). This multicomponent exercise training was scheduled twice a week (24 sessions over 12 weeks).

In addition, the app provided recommendations for endurance twice a week (24 sessions over 12 weeks), starting with 15 min (week 1) and gradually increasing to 45 min (week 12). Endurance recommendations followed aerobic training zones including rating of perceived exertion (5–6) (modified Borg CR10 Scale [1]). The app proposed sample aerobic activities for each session such as biking, swimming, cleaning windows or mowing the lawn, but participants were free to choose aerobic exercises that best suited their preferences.

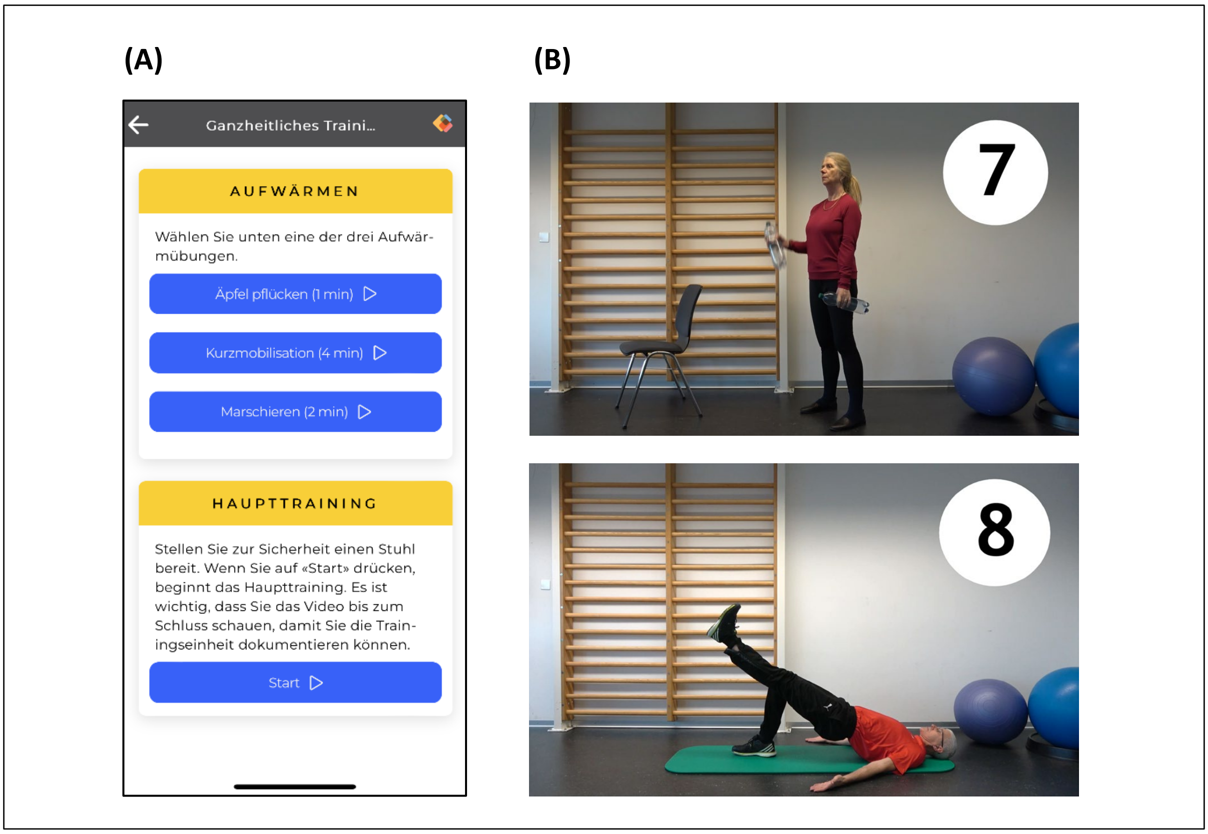

Figure 2: (A) Sample holistic training, (B – top) sample strength exercise level *beginner*, (B – bottom) sample balance exercise level *advanced* (Switzerland, 2024)

1.2. Nutrition

The nutrition domain offered information, advice and tips on nutrition in older age. It also included tasks, recipes and podcasts aimed at integrating appropriate dietary practices into daily routines. Two sessions per week (24 sessions over 12 weeks) of 5–25 min each were scheduled for this domain. Topics included nutrition-related changes in older age, eating patterns, energy requirements, proteins, minerals, vitamins, drinking, the plate model, enjoyable nutrition, food variety, and grocery shopping. Content was provided in written format and audio (Figure 3).

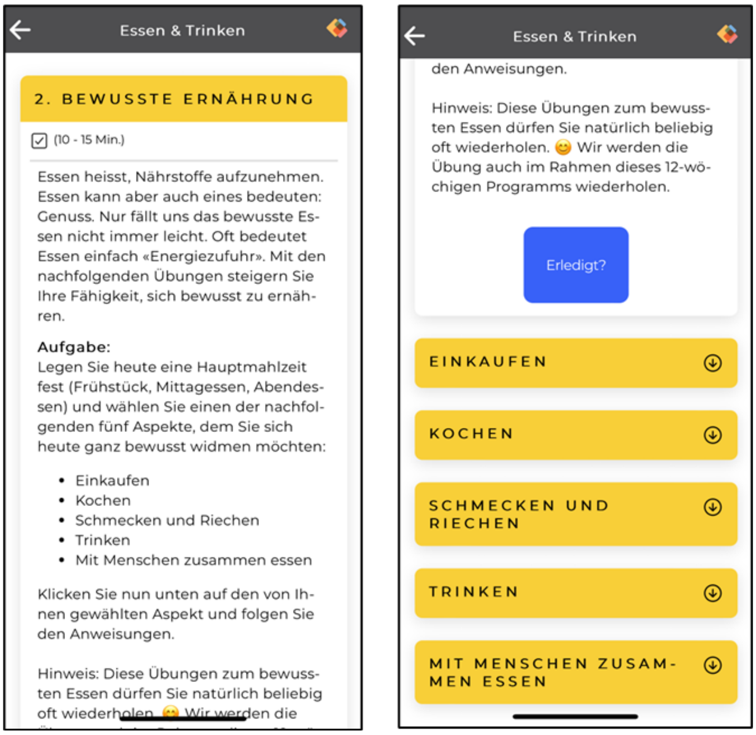

Figure 3: Sample session nutrition with introductory text, task and further information provided in drop-down boxes (Switzerland, 2024)

1.3. Sleep

The sleep domain provided knowledge, advice and guidance for improving sleep habits. Relevant topics for the target population were identified through available evidence and included factors that contribute to a healthy sleep, the purpose of sleep, age-related changes in sleep patterns, sleep duration, napping, waking during the night and the effects of caffeine, alcohol and nicotine on sleep. It also addressed common sleep-related issues in older age, including snoring, restless leg syndrome, nightmares and nocturia as well as the use of sleeping pills. The content extended to the relationship between sleep and other lifestyle domains. In addition, participants were asked to complete a sleep protocol for two weeks. The content was delivered through podcast episodes, with key messages from each episode summarized in written text in the app and the newsletter (Figure 4). Two sessions per week (12 sessions over 6 weeks) of 5–20 min each were scheduled for this domain.

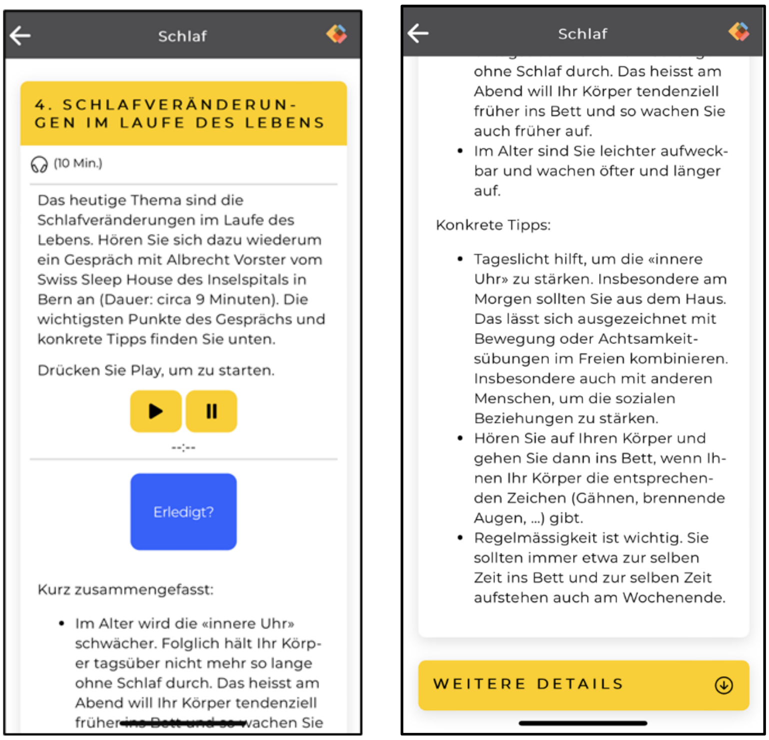

Figure 4: Sample session for the sleep domain with audio file (podcast) and summary of key messages in written format (Switzerland, 2024)

1.4. Mindfulness/relaxation

The mindfulness/relaxation domain introduced participants to evidence-based stress management techniques that have been shown to enhance well-being. We included three techniques: body scan, sitting meditation focusing on breath and progressive muscle relaxation. Each technique was practiced for two weeks. The participants were first introduced to each technique with a short video and were then provided with an audio file that guided them through each session (Figure 5). In addition, some theoretical background was provided as written text in the app and the newsletter. Two sessions per week (12 sessions over 6 weeks) of 20 min each were scheduled for this domain, but participants were informed that these techniques could be practiced more regularly.

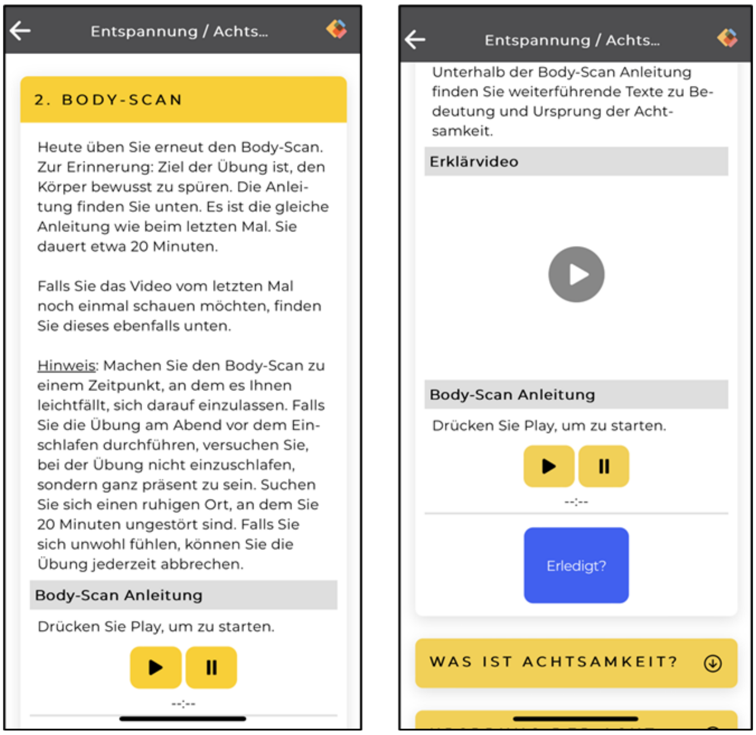

Figure 5: Sample session for mindfulness/relaxation with body scan audio file, introductory video and some additional information in drop-down boxes (Switzerland, 2024)

1.5. Newsletter

A weekly newsletter was part of the intervention, providing new lifestyle-related information while reinforcing previously covered content (Figure 6). The newsletter also explored topics such as cognitive training, nature and the environment, sexuality in older age, and dealing with death.

Newsletter Woche 8

DELIA

Bewegung

Können Sie Gläser noch gleich gut öffnen wie in jüngeren Jahren? Oder schwere Pfannen tragen? Die meisten von Ihnen werden das Nachlassen der Fingermuskulatur erlebt haben. Auch die Fussmuskulatur ist davon betroffen.
Im Rahmen unseres Programms haben Sie bereits einmal kennen gelernt, dass der menschliche Körper Muskeln abbaut und Bewegungen dadurch mühsamer werden. Sie haben aber auch erfahren, dass es nie zu spät ist mit dem Training zu beginnen. Denn bis ins hohe Alter kann das Muskelgewebe gestärkt werden. Dies trifft auch auf die Muskulatur der Hände und Füsse zu. Hier finden Sie beispielsweise ein paar gute Handgymnastik-Übungen.

Essen & Trinken

Diese Woche haben wir Sie über Abwechslung beim Essen informiert. So nehmen sie alle wichtigen Nährstoffe zu sich. Ihr Körper benötigt Energie in Form von Kohlenhydraten und Fetten, Proteine, Vitamine, Mineralstoffe und Wasser. Zudem kann abwechslungsreiches Essen Genuss bedeuten.

Schlaf

Im Bereich Schlaf haben Sie diese Woche erfahren, dass der Schlaf verschiedene lebenswichtige Funktionen für den gesamten Körper hat. Zudem haben Sie gelernt, dass im Alter die «innere Uhr» schwächer wird. Dadurch will Ihr Körper im Alter tendenziell früher ins Bett und Sie wachen früher auf. Ausserdem wachen Sie öfter und länger auf.
Tageslicht hilft, um die «innere Uhr» zu stärken. Deshalb sollten Sie insbesondere am Morgen aus dem Haus. Das lässt sich ausgezeichnet mit Bewegung oder Achtsamkeitsübungen im Freien kombinieren. Insbesondere auch mit anderen Menschen, um die sozialen Beziehungen zu stärken. Regelmässigkeit ist ebenfalls wichtig um die «innere Uhr» zu stärken. Deshalb sollten Sie immer etwa zur selben Zeit ins Bett und zur selben Zeit aufstehen.

Was ist gut für das Gehirn?

Ein Lebensstil mit ausreichend Schlaf, ausgewogener Ernährung, regelmässiger körperlicher Aktivität, tragenden soziale Beziehungen, genügend Entspannung, Achtsamkeit und Unabhängigkeit von Suchtmitteln trägt sehr viel zur Gesundheit Ihres Gehirns bei. Unser Programm bietet Ihnen also beste Voraussetzungen 😊.
Darüber hinaus empfehlen wir Ihnen regelmässiges Gehirntraining. Wir wissen heute, dass sich das Gehirn bis ins hohe Alter anpasst. Und ein anpassungsfähiges Gehirn lässt sich trainieren. Also, falls Sie Ihr Gehirn nicht bereits trainieren, starten Sie damit! Ein regelmässiges Gehirntraining trägt neben den anderen Lebensstilaspekten sehr viel zur Gesundheit Ihres Gehirns bei.

Sie können Ihre Gehirnzellen trainieren indem Sie:

- Täglich lesen (Zeitung, Zeitschriften, Bücher etc.)
- Denksportaufgaben lösen (Sudoku, Kreuzworträtsel etc., mittlerweile gibt es auch viele verschiedene Onlineangebote für «Gehirnjogging»)
- Anspruchsvolle Spiele spielen (z.B. Jassen oder Schach)
- Musizieren
- Tanzen
- Ihr Gehirn fordern, indem Sie beispielsweise eine neue Sprache lernen

Achten Sie darauf, dass Sie auch beim Gehirntraining Spass haben! Zudem tragen Kreativität und Humor zur Gesundheit Ihres Gehirns bei.

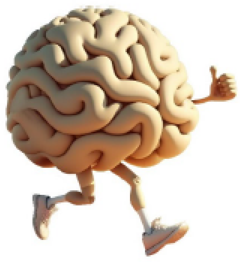

1

Figure 6: Sample newsletter (Switzerland, 2024)

1.6. Quiz

Weekly health and lifestyle quizzes were provided to reinforce knowledge acquisition.

DELIA

Quiz Woche 1

Anleitung: Lesen Sie die Aussage. Ist sie richtig oder falsch? Unten finden Sie die Auflösung.

**Bewegung**	
1	Ich muss mich mindestens 15 Minuten am Stück bewegen, um einen gesundheitlichen Nutzen zu haben.
2	Bewegung trägt nur zu meiner körperlichen Gesundheit bei.
3	Durch Bewegung kann ich positive Gesundheitseffekte bis ins hohe Alter erzielen, unabhängig davon, ob ich mich in jüngeren Jahren schon viel bewegt habe oder nicht.
**Essen & Trinken**	
4	Was und wie ich esse beeinflusst mein Wohlbefinden.
5	Essen ist purer Genuss.
6	Es gibt einen Ernährungstyp (z.B. «Ernährung ist Zwecksache»), der für alle Menschen am besten ist.
**Entspannung & Achtsamkeit**	
7	Stress kann sich körperlich, mental und/oder sozial auswirken.
8	Stress ist immer negativ.
9	Die Achtsamkeit fördert das bewusste Erleben von Augenblick zu Augenblick, ohne diese Augenblicke zu bewerten. Somit kann Achtsamkeit jederzeit im Alltag gelebt werden.
**Auflösung**	
1	Falsch. Jede Bewegung ist besser als keine Bewegung unabhängig von der Dauer. Somit gilt: Jede Bewegung zählt!
2	Falsch. Bewegung trägt zur körperlichen *und* mentalen Gesundheit bei. Und falls die Aktivität zusammen mit anderen Menschen stattfindet, auch zur sozialen Gesundheit.
3	Richtig. Es ist nie zu spät, um mit körperlicher Aktivität zu beginnen. Zudem gibt es keine Altersobergrenze, ab der keine positiven Effekte mehr auftreten.
4	Richtig. Essen & Trinken tragen zu Ihrem Wohlbefinden bei.
5	Für die einen Menschen stimmt diese Aussage, für andere nicht. Finden Sie heraus, welcher Ernährungstyp Sie sind.
6	Falsch. Essen & Trinken ist sehr individuell und Sie sollten herausfinden, wie Sie Ihr Wohlbefinden durch Essen & Trinken positiv beeinflussen können.
7	Richtig. Stress kann Sie auf verschiedenen Ebenen beeinflussen.
8	Falsch. In herausfordernden Situationen, die als lösbar empfunden werden, kann auch «positiver Stress» (genannt Eustress) auftreten.
9	Richtig. Achtsamkeit lässt sich sehr gut in den Alltag integrieren. Sei es beim Kaffeetrinken, Spazieren, Staubsaugen oder Kommunizieren mit anderen Menschen.

Figure 7: Sample quiz (Switzerland, 2024)

1.7. Personalization

There were several features of the app that were personalized. First, the weekly schedule was tailored to each user. Based on the user's preferences, the app featured a weekly schedule showing planned sessions from Monday to Sunday, with direct access to session content. Second, both the physical activity and nutrition domains were personalized.

The physical activity domain had three intensity levels: beginner, intermediate and advanced. The lowest level mainly comprised chair exercises. The highest level included floor exercises (e.g. side plank), where participants had to get down to the floor and stand up on their own. Two questions were used to determine the assignment of levels:

- "Are you able to lie down on the floor and easily get back up?" (yes or no)
- "How frequently have you engaged in strength training over the past year?" (never or rarely [less than once per month], occasionally [two to three times per month] or regularly [weekly])

The assignment algorithm for physical activity is shown in Table 1.

Table 1: Assignment algorithm for the three physical activity intensity levels (Switzerland, 2024)

**Ability to get on floor and stand up**		**Strength training (last year)**			**Level**
**Yes**	**No**	**< 1 per month**	**2-3 per month**	**Weekly**	
X		X			Beginner
X			X		Intermediate
X				X	Advanced
	X	X			Beginner
	X		X		Intermediate
	X			X	Intermediate

At the end of the first and sixth weeks, the app asked participants to rate the intensity of the training (too high, just right, too low). Based on their response, the intensity level was either adjusted or maintained.

The nutrition domain also operated on three levels/tracks: adiposity, normal weight and underweight/malnutrition. The assignment was based on three aspects:

- Current body mass index (BMI)
- Change in body weight over the past three months
- Food intake in the last week (unchanged/as usual [100%], slightly reduced [75-99%], reduced [50-75%], greatly reduced [25-49%], minimal to no intake [0-24%]); question adapted from the Nutritional Risk Screening (NRS) [2]

Based on this information, a preprogrammed algorithm assigned participants to a specific track (Table 2).

Table 2: Assignment algorithm for the three nutrition tracks (Switzerland, 2024)

**BMI**	**Assignment algorithm**
**>=30**	**Adiposity** track (regardless of answers to the other two questions)
**>=20-30**	**Normal weight** track<br><br>OR **underweight/malnutrition** track if:<br>• Weight change in last three months ≤ -10% OR food intake in last week 0-49%<br>• Weight change in last three months < -5% to -9% AND food intake last week 50-74%
**>=18.5-20**	**Underweight/malnutrition** track<br><br>OR **normal weight** track if:<br>• Weight change in last three months > -5% AND food intake last week >75%
**<18.5**	**Underweight/malnutrition** track (regardless of answers to the other two questions)

Although the content in the sleep and mindfulness/relaxation domain was not personalized based on information provided upon first login, the mindfulness/relaxation domain offered some flexibility. The audio files for each technique were made available to the users after introduction. While they were motivated to learn a new technique, they also had the opportunity to practice a previously introduced one. This approach allowed for both substitution and complementary use.

1.8. Additional features

The app included the following additional features to enhance user experience:

- Minimalistic app design using high contrast colours.
- Customizable font size: Participants could adjust the font size at any time.
- Diary: Participants could create diary entries at any time.
- Feedback and push notifications: The app automatically displayed motivational messages when participants entered data or completed sessions. In addition, push notifications were sent at key points each week: at the start (after completing the self-rated HRQoL assessment with the EQ VAS), midweek (as a reminder to continue using the app) and at the end (with the release of the weekly newsletter).
- Session completion: Participants could log completed sessions in the app, even if they completed them on a different day within the same week.
- Progress tracking: After each holistic training and endurance session, participants were asked to rate their perceived exertion using the modified Borg CR10 Scale [1] and record the session duration. These metrics could be tracked over 12 weeks.
- Support: Participants could reach out to the study team for assistance or inquiries via the app's contact form.
- Supplementary web access: Participants could access all resources, including videos, audio files, newsletters, and quizzes, through a simple web page secured by an access code.

1.9. Participant onboarding

The participants received all information regarding the intervention and study via email. This included detailed participant information, instructions on how to install the mobile application and a short overview of the main functions of the mobile application. In case of questions or issues, they could contact the study team via the contact form in the app or email.

At the beginning of the intervention, participants were informed that:

- The intervention intends to help be physically active on a regular basis, eat a healthy and balanced diet, improve sleep habits and boost mental well-being through relaxation/mindfulness exercises.
- The intervention guides on how to set goals, monitors progress and creates an individually tailored structured weekly schedule of activities based on the information provided at the beginning.
- The content is structured and compiled into an overall program. Therefore, it is recommended to view the content daily.
- The intervention can be used anytime and everywhere.
- The intervention lasts three months.
- The time required for the intervention varies from person to person, but it is approximately three to four hours per week.
- The intervention was specifically developed for people over 65 years of age involving older adults and various health professionals and scientists.
- The intervention does not replace any planned activities, such as primary care, nutritional counseling, psychological support or physiotherapy.

2. App development

The app development was primarily guided by the Integrate-Design-Assess-Share (IDEAS) framework [3] and followed an iterative participatory co-creation approach [4] following these key steps:

1. Cross-sectional survey study with older adults (phase 1 of IDEAS): At first a needs assessment for digital lifestyle interventions in Swiss community-dwelling older adults aged 60 years and above was conducted [5]. The goal was to investigate the needs, requirements and preferences for digital interventions to promote physical activity and additional lifestyle-related content in the target population. 922 respondents with a mean age of 72 years (SD 6.4, range 60–98) completed the online survey.
2. Semi-structured interviews and brainstorming sessions with service providers (phases 2–4 of IDEAS): Interviews with experienced health professionals and researchers from different lifestyle medicine disciplines including sleep, nutrition and dietetics, physiotherapy, exercise and sports science, mindfulness, psychology, gerontology, and software development were carried out to specify target behaviours, identify behavioural strategies, and generate ideas. All experts had experience working with older adults. Their input helped tailor our lifestyle intervention specifically for older adults.
3. Prototype (phase 5 of IDEAS): Based on the previous steps, we developed an initial mock-up of the multidomain lifestyle app.
4. Mock-up testing with older adults (phase 6 of IDEAS): The mock-up was tested with six older adults from the patient advisory board of the University Hospital of Bern. They provided valuable feedback on the app's interface, navigation, and usability.
5. Minimum viable product (phase 7 of IDEAS): Based on the previous steps, we built a fully functional minimum viable product with the most essential features.
6. Pilot testing with older adults and service providers (phase 8 of IDEAS): Once all app features were implemented, we tested its functionality with project team members during a one-week testing phase. Subsequently, we performed a pilot test with six older adults. Their two-week testing experience yielded valuable insights that we used to further improve the app.

3. Interview guide

Interview guide (developed based on literature as well as pre- and post-intervention questionnaire data) for individual interviews with senior citizens

Introduction:

1. Welcome and introduction of the interviewer
2. Expression of gratitude for the participant's availability and time
3. Announcement of the approximate interview duration (30min)
4. Explanation of the study objective:
   "The study aims to investigate the use and usability of a mobile application with a focus on physical activity, nutrition, sleep, mindfulness/relaxation. Today's interview aims to gather your perspective on the application and its potential for further development."
5. Clarification of participant rights:
   a. Confidentiality, data anonymization and exclusive use within the study
   b. Voluntary participation
   c. Option to terminate participation at any time
   d. Recording of the interview, with deletion upon completion of the analysis
6. Start of the interview and recording

Topics	Main question	Follow-up question	Maintenance question
**Introduction/general questions about the app**	1. Please tell us what you liked best about the app and what you liked least.<br>2. Why did you decide to take part in testing the app?<br>3. In what ways were your expectations met and in what ways were they not?<br>4. How motivating was the app to try something new?		
You have tested an app in the last 12 weeks with a focus on four lifestyle domains, namely physical activity, nutrition, sleep, mindfulness/relaxation.<br>In the interview, I will ask general questions about the app, but also go into the individual lifestyle domains.			

**Physical activity**	Now think about the "physical activity" domain, where you have been given the opportunity to perform holistic training twice a week (videos) and an endurance session twice a week. In addition, the weekly newsletters and quizzes contained information related to exercise.<br><br>1. What content in the physical activity domain was helpful and why? What content in the physical activity domain was not helpful and why?<br><br>2. Thinking about the last 12 weeks, how have the topics covered in the app influenced your physical activity behaviour?<br><br>3. How will you adapt your everyday life regarding physical activity in the future?<br>How could the app support you?	a) How helpful were the videos? How helpful were the endurance training instructions? How interesting were the texts you read (newsletters and quizzes)?	• Can you give some examples?

**Nutrition**	Now think about the "nutrition" domain. In this lifestyle domain, you read texts, listened to podcasts and completed tasks twice a week.<br><br>1. What content in the nutrition domain was helpful and why?<br>What content in the nutrition domain was not helpful and why?<br><br>2. When you think about the last 12 weeks, how have the topics covered in the app influenced your eating habits?<br><br>3. How will you adapt your daily diet in the future?<br>How could the app support you?	a) How interesting were the conversations you heard or the texts you read (in the app itself or the newsletters and quizzes)?	• Can you give some examples?

**Sleep**	Now think about the "sleep" domain, where you listened to podcasts twice a week and received a short summary of each podcast to read. There were also tasks to solve.<br><br>1. What content in the sleep domain was helpful and why?<br>What content in the sleep domain was not helpful and why?<br><br>2. Thinking about the last 12 weeks, how have the topics covered in the app affected your sleep?<br><br>3. how will you adapt your everyday life regarding sleep in the future?<br>How could the app support you?	a) How interesting were the conversations you heard or the texts you read (in the app itself or the newsletters and quizzes)?<br><br>b) How interesting were the tasks, e.g. sleep diary?	• Can you give some examples?

**Mindfulness/relaxation**	Now think about the "mindfulness/relaxation" domain. There were texts to read, introductory videos on mindfulness/relaxation techniques and exercise instructions. You could do the exercises twice a week.<br><br>1. What content in the mindfulness/relaxation domain was helpful and why?<br>What content in the mindfulness/relaxation domain was not helpful and why?<br><br>2. Thinking about the last 12 weeks, how have the topics covered in the app affected your sense of well-being?<br><br>3. How will you adapt your everyday life in the future with regard to dealing with stress/tension and mindfulness/relaxation?<br>How could the app support you?	a) How helpful were the introductory videos?<br>How helpful were the exercise instructions?<br>How interesting were the texts you read (newsletters and quizzes)?	• Can you give some examples?

**Diary**	Did you keep the diary in the app?	If YES: What did you record in the diary and why?<br>If NO: why not?	• Did you intend to communicate something to us (the project team) with the diary entries or did you keep it for yourself?
**Development potential**	We would like to adapt and improve the app in the future.<br>a) Where do you see potential for improvement to better meet your needs?	a. Are there areas where the use of the app should be further developed?<br><br>b. Are there areas that would be better carried out analog (i.e. face-to-face) than on an app?<br><br>c. If you could, would you continue to use the app?<br><br>d. Would you recommend the app to others?<br><br>e. Have you used the website and if yes, for what? If no, why not?<br><br>f. Could you imagine using this app in the outdoors, i.e. sitting on a	• Can you describe ideas on what this could look like?<br>• Which areas did you miss in the program?<br>• Was there anything that bothered you about the app?<br><br>• If NO: why?<br><br>• if YES: why?<br>• if NO: why?

		park bench and reading the newsletters or listening to the audio, and possibly doing some of the exercises?	
**Conclusion**	b) Are there any **other aspects** that you consider relevant in this context and would like to share with us?		

Outro:

1. Expression of gratitude for participation
2. Notification of the end of the recording

4. Additional results

4.1. Baseline characteristics of the study population

Physical activity

We assessed self-reported physical activity at pre-intervention using a modified version of the Godin-Shephard Leisure-Time Physical Activity Questionnaire to get a better understanding of the study population [6]. The Leisure Score Index (LSI) was estimated using the following formula [6]: LSI=(frequency of light×3)+(frequency of moderate×5)+(frequency of strenuous×9). This formula uses weights based on metabolic equivalents to calculate the LSI score. The LSI at pre-intervention was on average 53.9 (SD 35.4). The mean number of times of strenuous physical activity per week was 2.2 (SD 2.4) and the mean number of minutes per week was 146.5 (SD 238.5; median 60). For moderate physical activity, the mean number of times per week was 4.1 (SD 2.9) and the mean number of minutes per week was 225.6 (SD 267.9; median 150). The mean number of times of mild physical activity per week was 4.6 (SD 3.3) and the mean number of minutes per week was 231.9 (SD 243.4; median 165).

Regarding the three different intensity levels of the holistic training: 48.5% of the participants started with the beginner level, 25.2% with the intermediate level and 26.3% with the advanced level. Among the participants, 26.3% reported engaging in strength training weekly, 25.3% two to three times per month and 48.5% less than once per month. Additionally, 6.1% reported difficulty getting down to the floor easily.

Nutrition

We assessed self-reported nutritional aspects at pre-intervention using the questions from the latest Swiss Health Survey [7]. Among the participants, 78.8% reported eating all types of food, 7.1% reported not eating fish, 13.1% reported not eating meat and one participant reported following a vegan diet. The mean number of days per week with vegetable consumption was 6.3 (SD 1.1) and the mean number of portions per day was 2.2 (SD 1.3). The mean number of days per week with fruit consumption was 5.7 (SD 2.0) and the mean number of portions was 1.8 (SD 1.1). On average, participants reported to eat meat on 2.9 days per week (SD 1.9) and fish on 0.9 days per week (SD 0.8). Sweets were consumed on average on 4.4 days per week (SD 2.2) and the mean number of portions per day was 1.6 (SD 1.3). Salty snacks were consumed on average on 1.5 days per week (SD 1.6) and the mean number of portions per day was 1.6 (SD 1.3). The mean number of days per week with sugar-sweetened beverage consumption was 0.6 (SD 1.4) and the mean litres per day was 0.4 (SD 0.4). Alcohol was consumed on average on 2.5 days per week (SD 2.2).

Regarding the three different tracks in the nutrition domain: 11.2% were assigned to the adiposity track, 81.6% to the normal weight track and 7.1% to the underweight/malnutrition track. Based on self-reported data, 6.1% of the participants had a BMI at pre-intervention smaller than 18.49, 5.1% between 18.5 and 19.99, 77.6% between 20 and 29.99 and 11.2% bigger or equal to 30. Change in body weight in the last three months was ≤ -10% in 2.0% of participants, > -10% and ≤ -5% in 5.1% and > -5% in 92.9%. Food intake in the last three months before the intervention was unchanged/usual in 89.8 of the participants, slightly reduced in 9.2% and reduced in 1.0% of the participants. None of the participants reported a greatly reduced food intake or minimal to no intake.

Sleep

Self-reported sleep behaviour and issues were assessed at pre-intervention using the Bernese Sleep Health Questionnaire [8]. The mean sleep duration was 7.0 hours (SD 1.1; range 4 – 9 hours). The mean time spent in bed was reported to be 7.9 hours. Problems falling asleep or being awake more than 30 minutes during the night at least once per week were reported by 38.0% of the participants. Napping at least once per week was reported by 28.7%. Restless legs syndrome at least once a week was reported by 11.1% and 0.9% reported having nightmares at least once a week. 6.5% reported taking at least once a week sleeping pills.

4.2. Weekly EQ VAS

The weekly EQ VAS scores of participants finishing the twelve week intervention are presented in Figure 8.

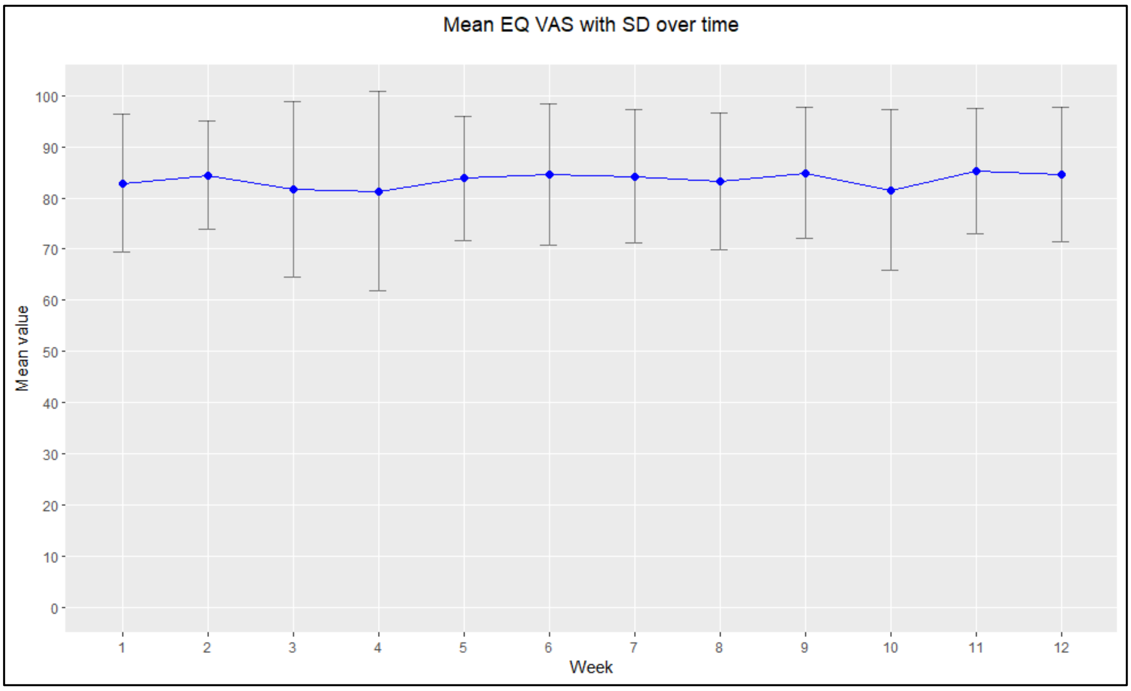

Figure 8: Mean EQ VAS scores with standard deviation (SD) throughout the intervention period (n=57) (Switzerland, 2024)

4.3. Non-completers versus completers – Additional aspects

In the subsample who stopped using the app before week twelve, the average score was lower for SF-36 mental health (76.8 vs. 79.7), SF-36 vitality (65.7 vs. 79.7), the flourishing scale (46.3 vs. 47.1) and satisfaction of life scale (27.6 vs. 27.7). The percentage being at least somewhat satisfied with their current lifestyle behaviour was lower for the physical activity domain (72.9% vs. 88.3%), nutrition (81.3% vs. 90.0%) and mindfulness/relaxation (70.8% vs. 75.0%) but higher in the sleep domain (79.2% vs. 71.7%). Readiness to change was lower for physical activity (8.1 vs. 8.3), sleep (7.1 vs. 7.8) and mindfulness/relaxation (7.6 vs. 8.3) but higher for nutrition (7.9 vs. 7.7).

4.4. Newsletter and quiz

Respondents of the post-intervention questionnaire reported having read a high number of newsletters, with a mean of 10.9 out of 12 newsletters (SD 2.3). Nine out of fifteen interviewees considered the newsletters helpful because they *confirmed what was already known* (four interviewees), *provided a repetition of important aspects in a compact and informative way* (three interviewees), and *could be printed out* (two interviewees).

The self-reported number of quizzes completed was also high, with a mean of 9.4 out of eleven quizzes (SD 3.1). However, the interviewees reported mixed experiences with the quizzes. On the one hand, they were perceived as *informative, good, entertaining and as a confirmation of what they had read* (four interviewees). On the other hand, they were perceived as *too simple and not motivational* (three interviewees).

4.5. App usability (MAUQ) – Detailed results

The ratings for each question of the MAUQ are presented in Table 3.

Table 3: Ratings for each single question of the MAUQ (n=57) (Switzerland, 2024)

	Mean	SD	Median	Min	Max
**Ease of use**					
The app was easy to use.	6.4	0.7	7	4	7
It was easy for me to learn to use the app.	6.4	0.9	7	2	7
The navigation was consistent when moving between screens.	6.0	1.1	6	3	7
The interface of the app allowed me to use all the functions (such as entering information, responding to reminders, viewing information) offered by the app.	5.9	1.1	6	3	7
Whenever I made a mistake using the app, I could recover easily and quickly.	5.2	1.3	5	2	7
**Interface and satisfaction**					
I like the interface of the app.	5.7	1.0	6	2	7
The information in the app was well organized, so I could easily find the information I needed.	6.1	1.0	6	3	7
The app adequately acknowledged and provided information to let me know the progress of my action.	5.3	1.3	6	2	7
I feel comfortable using this app in social settings.	5.5	1.5	6	1	7
The amount of time involved in using this app has been fitting for me.	6.0	0.9	6	3	7
I would use this app again.	5.7	1.5	6	1	7
Overall, I am satisfied with this app.	6.0	1.0	6	2	7
**Usefulness**					
The app would be useful for my health and well-being.	5.2	1.5	6	1	7
The app improved my access to health care services	5.0	1.4	5	1	7
The app helped me manage my health effectively.	5.2	1.4	6	1	7
This app has all the functions and capabilities I expected it to have.	5.4	1.3	6	2	7
I could use the app even when the Internet connection was poor or not available.	4.1	1.6	4	1	7
This mHealth app provided an acceptable way to receive health care services, such as accessing educational materials, tracking my own activities, and performing self-assessment.	5.5	1.3	6	2	7

Note. SD, standard deviation; Min, minimum; Max, maximum

4.6. Pre-post comparison of effectiveness measures – Detailed results

Pre- to post-changes of the outcome measures are presented in Table 4. Analyses for each single item contributing to the compound outcome measure for SF-36 mental health are presented in Figure 9, for SF-36 vitality in Figure 10 and the flourishing scale in Figure 11.

Table 4: Pre- to post-changes in outcome measures of participants using the app for twelve weeks (n=57) (Switzerland, 2024)

**Outcome measure**	Pre, mean (95% CI)	Post, mean (95% CI)	Mean change (95% CI)	P value
**SF-36 mental health**	79.8 (76.6-83.0)	82.8 (80.4-85.2)	3.0 (0.8-5.2)	0.028
**SF-36 vitality**	69.2 (65.6-72.8)	72.3 (69.6-75.0)	3.1 (0.0-6.1)	0.161
**Flourishing scale**	46.8 (45.7-48.0)	47.9 (46.7-49.1)	1.1 (0.3-1.8)	0.021

Confidence Interval (CI)

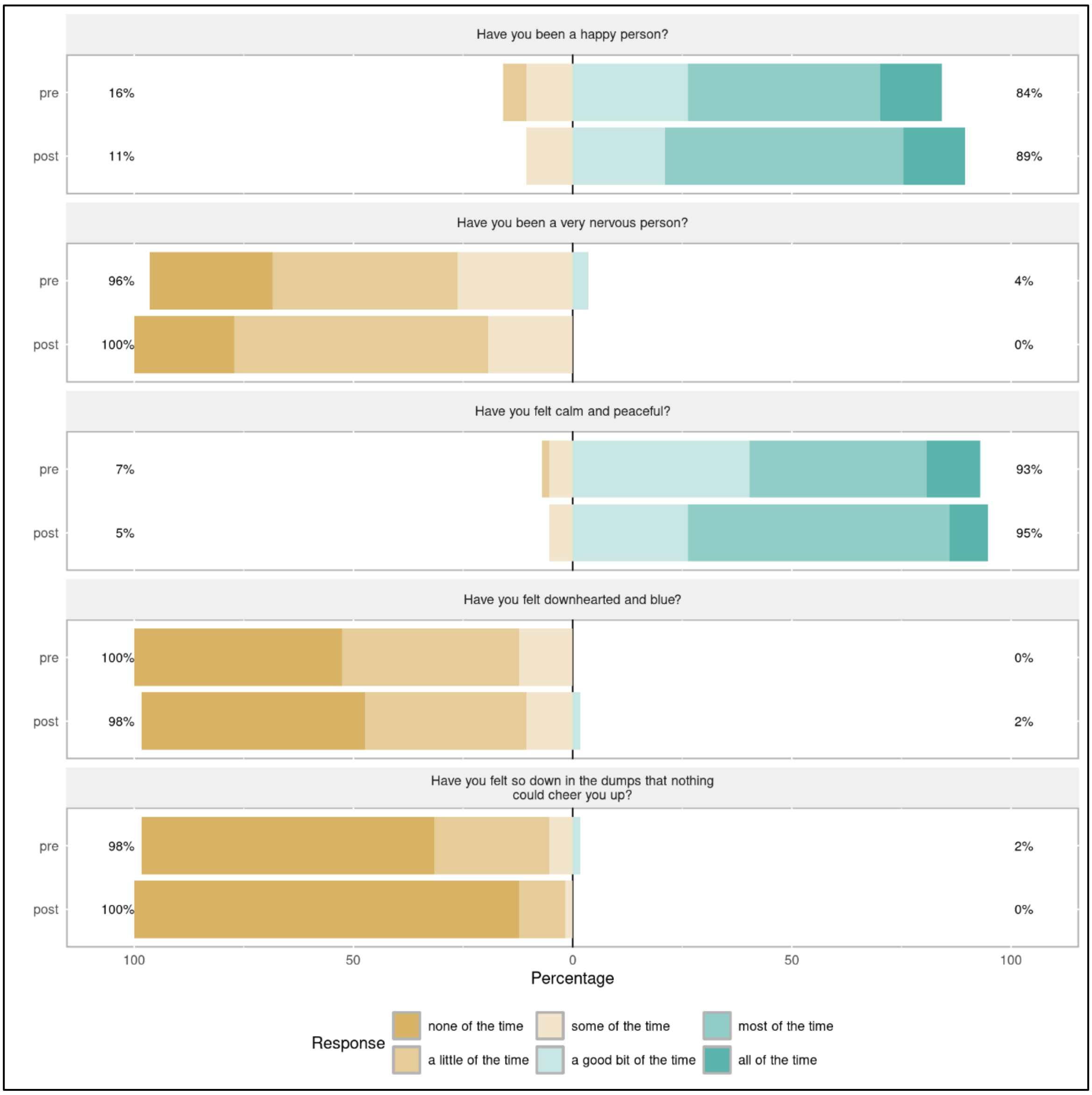

Figure 9: Pre-post comparison of SF-36 mental health single items (n=57) (Switzerland, 2024)

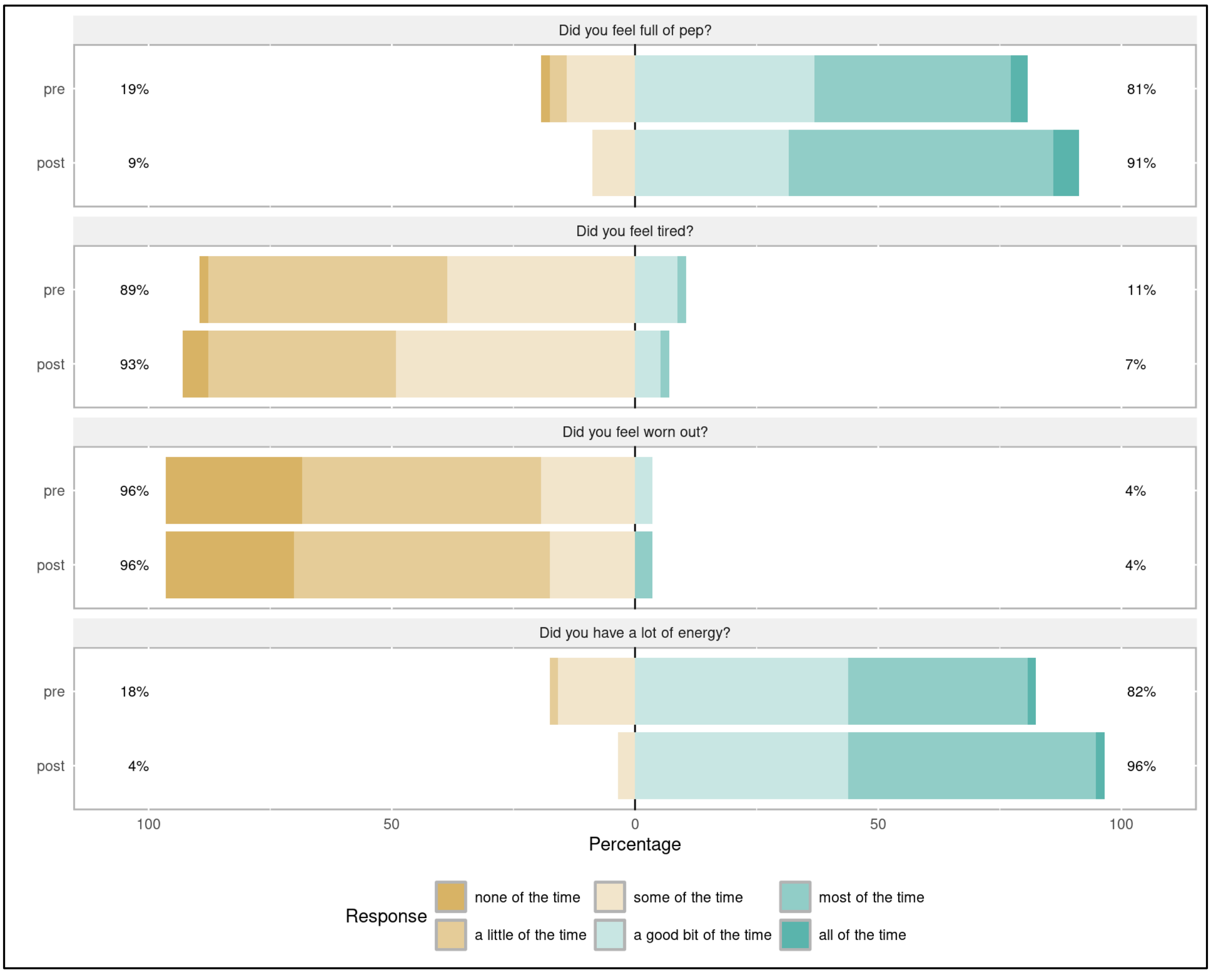

Figure 10: Pre-post comparison of SF-36 vitality single items (n=57) (Switzerland, 2024)

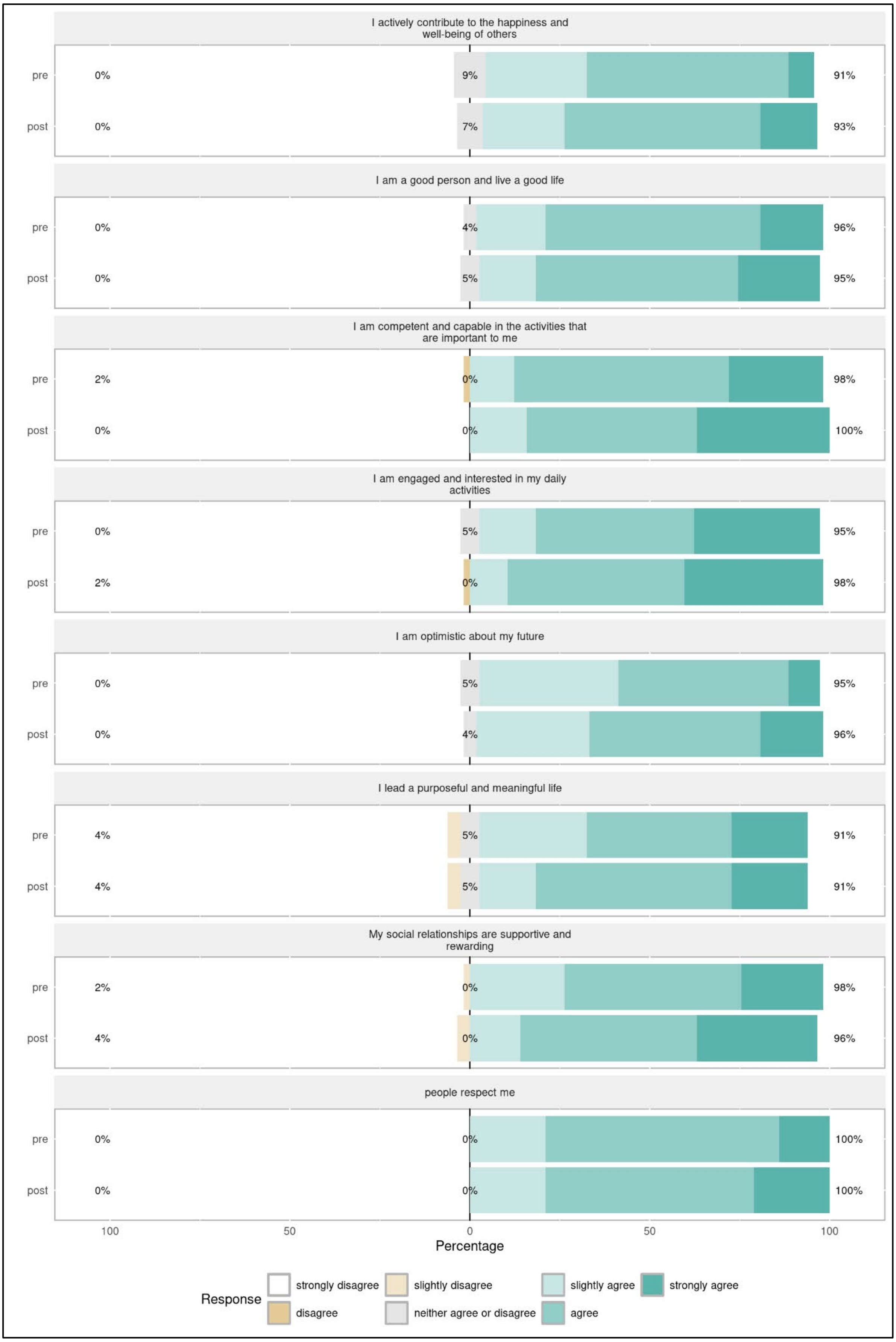

Figure 11: Pre-post comparison of flourishing scale single items (n=57) (Switzerland, 2024)

5. References

1. Borg, G. *Borg's perceived exertion and pain scales*. Champaign, IL, US: Human Kinetics, 1998

2. Kondrup, J, Rasmussen, HH, Hamberg, O and Stanga, Z. Nutritional risk screening (NRS 2002): a new method based on an analysis of controlled clinical trials. *Clin Nutr* (2003) 22: 321–336 . doi: 10.1016/S0261-5614(02)00214-5

3. Mummah, SA, Robinson, TN, King, AC, Gardner, CD and Sutton, S. IDEAS (Integrate, Design, Assess, and Share): A Framework and Toolkit of Strategies for the Development of More Effective Digital Interventions to Change Health Behavior. *J Med Internet Res* (2016) 18: e317 . doi: 10.2196/jmir.5927

4. Leask, CF, Sandlund, M, Skelton, DA, Altenburg, TM, Cardon, G, Chinapaw, MJM, et al. Framework, principles and recommendations for utilising participatory methodologies in the co-creation and evaluation of public health interventions. *Res Involv Engagem* (2019) 5: 2 . doi: 10.1186/s40900-018-0136-9

5. Weber, M, Schmitt, K-U, Frei, A, Puhan, MA and Raab, AM. Needs assessment in community-dwelling older adults toward digital interventions to promote physical activity: Cross-sectional survey study. *Digit Health* (2023) 9: 20552076231203785 . doi: 10.1177/20552076231203785

6. Godin, G. The Godin-Shephard Leisure-Time Physical Activity Questionnaire. *Health Fit J Can* (2011) 4: 18–22 . doi: 10.14288/hfjc.v4i1.82

7. Federal Statistical Office. Schweizerische Gesundheitsbefragung., https://www.bfs.admin.ch/bfs/de/home/statistiken/gesundheit/erhebungen/sgb.html#1243813114 (2023, accessed 3 March 2023)

8. Vorster, APA, van Someren, EJW, Pack, AI, Huber, R, Schmidt, MH and Bassetti, CLA. Sleep Health. *Clin Transl Neurosci* (2024) 8: 8 . doi: 10.3390/ctn8010008
